# The application of tissue engineering strategies for uterine regeneration

**DOI:** 10.1016/j.mtbio.2025.101594

**Published:** 2025-02-18

**Authors:** Shangsi Chen, James J. Yoo, Min Wang

**Affiliations:** a Department of Mechanical Engineering, Faculty of Engineering, The University of Hong Kong, Pokfulam Road, Hong Kong; b Wake Forest Institute for Regenerative Medicine, Wake Forest University Health Sciences, Medical Center Blvd, Winston-Salem, NC, 27157, USA

**Keywords:** Tissue Engineering, Uterine Regeneration, Stem Cell, Biomolecule, Biomaterial

## Abstract

Uterine injuries, particularly damages to endometrium, are usually associated with abnormal menstruation, recurrent miscarriage, pregnancy complications, and infertility. Tissue engineering using cell-based, biomolecule-based, or biomaterial and scaffold-based strategies has emerged as a novel and promising approach for uterine regeneration. Stem cells, biomolecules, and porous scaffolds used alone or, very often, used in combination as a more effective treatment means have shown great potential in promoting uterine regeneration. The reported preclinical studies have indicated that appropriate tissue engineering strategies could safely and effectively reconstruct not only endometrium but also partial or even the whole uterine structure. However, the progress in the uterine regeneration area is slow in comparison to that of regenerating many other body tissues and hence it still remains a great challenge to apply uterine tissue engineering for clinical applications. In this review, conventional treatments for uterine-related diseases are briefly reviewed and discussed first. Subsequently, tissue engineering strategies (cell-based, biomolecule-based, biomaterial and scaffold-based, or their combinations) for uterine repair in preclinical studies and clinical trials are presented and analyzed. Finally, the challenges and perspectives in uterine regeneration are pointed and discussed. Despite various limitations and obstacles, the tissue engineering approach is viable and holds high promise for uterine regeneration.

## Graphical abstract

**Figure:**
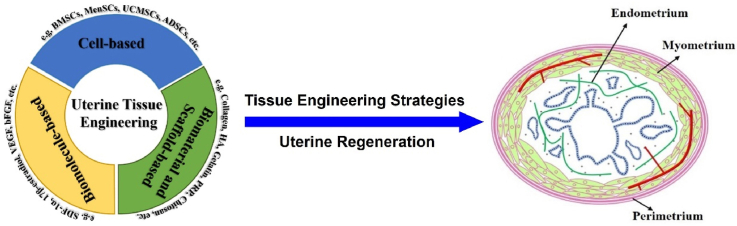

## 1 Introduction

The World Health Organization (WHO) defines infertility as the inability to achieve clinical pregnancy after 1-year regular unprotected sexual intercourse [1]. Around the world, 8–10 % of couples at the reproductive age (roughly from 12 to 49 years old) face the infertility problem [2]. Previous data from the United States Centers for Disease Control and Prevention indicated that in USA, approximately 1.5 million female patients aged from 15 to 44 years were medically diagnosed for infertility [3]. Up to 38 % of infertility are due to the ovulatory disorder and endometriosis, which can be well treated clinically. However, many other female patients suffer from uterine-related diseases that cannot be treated well clinically [4]. Among them, many women have been diagnosed for absolute uterine factor infertility (AUFI) because of the dysfunction of uterus. The causes of AUFI may be congenital (e.g., Mayer-Rokitansky-Küster-Hauser syndrome, uterine hypoplasia, uterine malformation) or acquired [e.g., gynecologic cancers, leiomyoma, adenomyosis, postpartum hemorrhage, and intrauterine adhesions (IUAs)] [5,6].

Generally, gynecologic cancers and severe IUAs have been regarded as two representative causes of female infertility. According to American Cancer Society, 15–20 % of cancers suffered by women are gynecologic cancers [7]. Although most of gynecologic cancers occur in postmenopausal females, there are still about 20 % in women of the reproductive age. Particularly, 8 % of endometrial, 12 % of ovarian, and 40 % of cervical cancers occur in the child-bearing age [8]. Consequently, many patients would impair or even lose their fertility potential during their cancer treatment. On the other hand, IUAs, also referred to as Asherman's Syndrome, is a very common disease in women of the fertile age. The first description of IUAs was reported by Fritsch in 1894 [9]. Later in the 1950s, Asherman revealed the history of 29 women with amenorrhea secondary to trauma of uterine cavity, i.e., the Asherman's Syndrome [10]. Currently, there are multiple classification systems to distinguish the severity of IUAs based on different mechanisms. A commonly used classification system provided by the American Society for Reproductive Medicine (ASRM) stipulates the severity of IUAs based on the extent of cavity involvement, in which mild, moderate, and severe IUAs are defined [11]. The occurrence of IUAs mainly results from the physical/mechanical damage, for example, mechanical manipulations including dilation and curettage (D&C) and hysteroscopy, to the uterine endometrium [12,13]. IUAs in moderate and severe levels could induce narrow uterus cavities and therefore cause infertility.

Organ transplantation has been recognized as the gold standard to treat the majority of organ damages and dysfunctions. Nowadays, allogenic uterus transplantation has also been popularly applied to help female patients with dysfunctional uterus for restoring fertility [[14], [15], [16]]. For instance, the first acknowledged human uterus transplantation attempt occurred in 2000 when a surgeon successfully transplanted a uterus taken from a 46-year-old patient into a 26-year-old woman [17]. Later, Brännström *et al.* provided a proof-of-concept about the first-time pregnancy after allogeneic uterus transplantation in rats [18]. Then, Brännström and his colleagues reported the first clinical livebirth after uterus transplantation in 2015 in female patient [19]. Afterward, the successful pregnancy and livebirth have been increasingly reported after uterus transplantation [[20], [21], [22], [23]]. Although applying allogenic uterus transplantation to treat AUFI has gained great progress, there are still some inevitable problems and limitations. The shortage of donors, possible diseases transmission, and immune rejection are the key factors to hinder the process of uterus transplantation. A few failure cases have been reported worldwide due to the immune rejection and the need for long-term immunosuppression [24,25]. Therefore, a novel strategy is needed in urgency. Fortunately, tissue engineering and regenerative medicine has emerged as a promising approach for the treatment of tissue damage, degeneration, and dysfunction [26].

Since the end of last century, the application of tissue engineering strategies for treating various tissues defects, such as bone [27,28], cartilage [29,30], skin [31,32], neuron [33,34], heart [35,36], liver [37,38], kidney [39,40], etc., has been flourished and made great progress. The general idea for tissue engineering is to construct three-dimensional (3D) scaffolds with interconnected pores in combination with specialized living cells and biomolecules to restore, maintain, or improve the original and unique structure and functions of damaged tissues or organs [26,41]. 3D porous scaffolds can provide physical/biochemical support for living cells [28]. Biomolecules, including growth factors, hormones, cytokines as well as chemokines, incorporated in scaffolds can modulate cell behaviors, such as attachment, migration, proliferation, and differentiation and therefore facilitate tissue regeneration [42,43]. Although tremendous success has been made in repairing various tissue defects, the application of tissue engineering strategies for uterine regeneration stayed lagged [44]. At present, limited studies have applied tissue engineering approach to reconstruct the structure and functions of damaged uterine tissues [5,[45], [46], [47]]. In general, IUAs and endometrium regeneration are the primary focus of current studies, and the scientists are bound up in regenerating the functions of endometrium but ignoring the function of myometrium. In consequence, the investigations of constructing the whole structure of the mammalian uterus are rare. However, owing to the rapid economic and social development, more and more interests are concentrating on the uterine regeneration. Furthermore, applying tissue engineering strategies for repairing damaged uterine tissues and restoring fertility has shown great potential and become more and more popular.

**Fig. 1:**
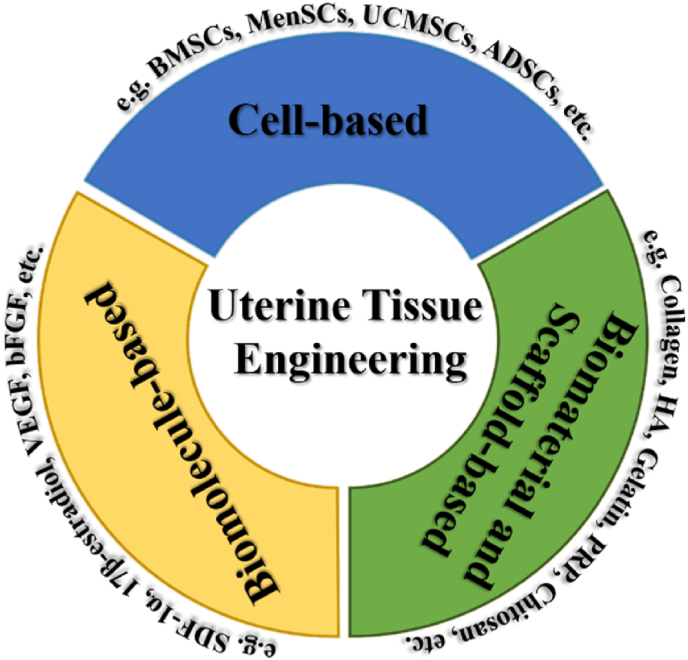
Cell-based, biomolecule-based, and biomaterial and scaffold-based tissue engineering strategies for uterine regeneration.

In this review, tissue engineering strategies for uterine regeneration will be introduced, summarized, analyzed, and separated into three parts: cell-based, biomolecule-based, biomaterial and scaffold-based (Fig. 1). The *in vivo* studies and clinical applications of tissue engineering strategies for repairing damaged uterine tissues and restoring fertility will be further explored. Finally, the limitations and future perspectives in uterine tissue engineering will be discussed.

## 2 Structure and functions of human uterine tissues

**Fig. 2:**
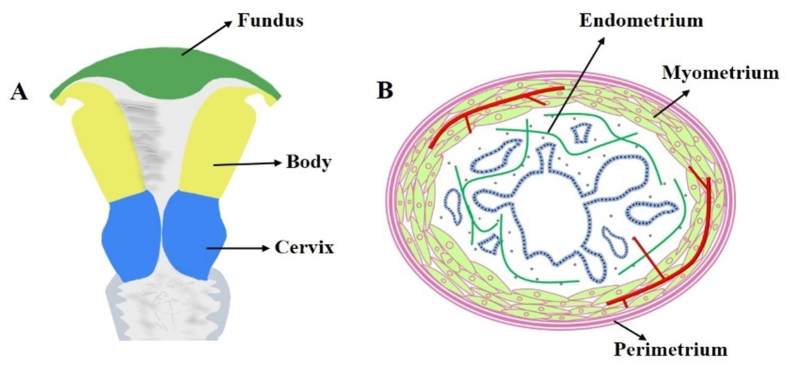
Structure of the uterus: (A) Front view (schematic) (B) Cross-sectional view (schematic), (C) Cross-sectional view.

Human uterus is a complex and dynamic organ with hierarchical structure and supports many essential biological functions in reproduction, i.e., implantation and development of embryos [48]. As shown in Fig. 2, the uterus exhibits a pear-shaped morphology and is characterized by its thick muscular walls (7.0 × 5.0 × 2.5 cm). This anatomical feature enables the uterus to accommodate and withstand substantial expansion and contraction during fetal development and parturition. The location of the uterus in the mammalian body is connected distally to the vagina, and laterally to uterine tubes. The uterus can be anatomically divided into three distinct regions: the fundus, body, and cervix. The fundus represents the uppermost part of the uterus situated above the uterine tubes. The segment of the uterus located superior to the isthmus is referred to as the uterine body, while the inferior portion is known as the cervix. Typically, implantation of a blastocyst occurs within the uterine body.

**Fig. 3:**
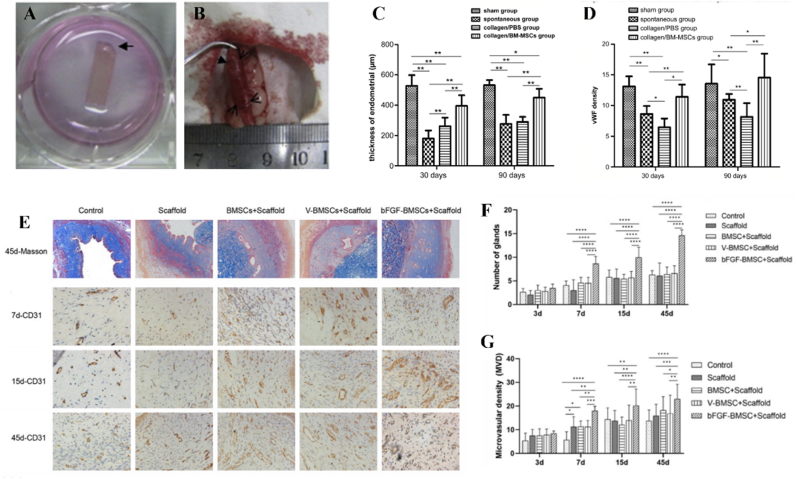
The transplantation of collagen/BMSCs scaffolds for uterine regeneration. (A) Collagen scaffolds loading BMSCs cultured in cell medium. (B) A segment of uterine tissues of 1.5 cm in length and 0.5 cm in width was cut and removed for collagen/BMSCs scaffolds implantation. (C) The endometrium thickness of collagen/BMSCs, collagen/PBS, spontaneous and sham group after 30- and 90-days operation. (D) The expression of von Willebrand factor (vWF) of collagen/BMSCs, collagen/PBS, spontaneous and sham group after 30- and 90-days operation [139]. The transplantation of collagen/bFGF-transfected BMSC scaffolds for uterine regeneration. (E) Masson staining results and the expression of CD31 of uterine tissues after treated by collagen/bFGF-transfected BMSC scaffolds. (F) The number of gland and (G) microvascular density of uterine tissues after implantation of collagen/bFGF-transfected BMSC scaffolds for 45 days in rat [132].

In addition, the cross-section of uterine fundus and body are composed of three tissue layers (Fig. 2, Fig. 3). The outer layer terms as perimetrium, also called peritoneum, which is a double-layered membrane. The fundamental function of perimetrium is to protect the uterus from friction by secreting watery serous fluid from a smooth layer of squamous epithelium on its surface. The perimetrium is made up of superficial mesothelium, and a very thin layer of loose connective tissue is beneath it. The middle tissue layer is called myometrium, which mainly comprises smooth muscle cells. The main function of myometrium is to induce uterus contraction and expansion, which is extremely important for fetus development and parturition. Additionally, the myometrium supports stromal and vascular tissue. The most important inner layer is recognized as endometrium. The human endometrium performs a remarkable regenerative capacity, and it can grow 4–6 mm within 5–6 days after menstruation cycle. At present, the clinical trials have revealed that a thin endometrium thickness (<7 mm) might impact the fertility capacity of women, thus resulting in a low pregnant rate [49]. The endometrium consists of a single-layered prismatic epithelium with or without cilia (depending on how far along the menstruation cycle is) and its basal lamina, uterine glands, and a specialized, cell-rich connective tissue (stroma) containing a rich supply of blood vessels. The structure of endometrium can be further divided into two layers: the basalis layer and functionalis layer [50]. The basalis layer is adjacent to myometrium and below the functionalis layer. It is intact and consists of stromal tissue and uterine glands. The stromal tissue is the major source of endometrium stem cells. The functional layer is the outer layer of the uterus. The functional layer undergoes degeneration, growth, and reorganization throughout the whole menstrual cycle. The endometrial glands are mainly located at the endometrium functional layer. The endometrium glands synthesize, secret and transport cytokines and growth factors, which are essential for survival and development of the embryos and fetus.

Rodents, such as rats and rabbits, possess a duplex uterus characterized by completely separated uterine horns, each with its own cervix that opens into the vagina. Specifically, rats have a bicornuate uterus consisting of right and left horns, allowing them to carry multiple offspring. Similarly, the rodent uterus, like that of other mammals, comprises three primary layers: the endometrium, myometrium, and perimetrium. A key difference between human and rodents lies in the uterus's shape and structure: humans have a single, pyriform uterus, while rodents have a bicornuate uterus with multiple implantation sites in each uterine horn. Additionally, the myometrium in rodents has a clearly defined outer longitudinal layer and inner circular layer, whereas in humans, these layers are less distinct.

## 3 Conventional treatments for uterus-related diseases

Indeed, IUAs and gynecologic malignancies such as uterine leiomyomas, commonly referred to as fibroids or myomas, are two significant concerns impacting patients' overall health and reproductive potential. Traumas and damages caused by surgical and mechanical interventions, which primarily injure endometrium inner layer, are the main reasons for the occurrence of IUAs. In circumstances of severe IUAs, connective tissues including collagen fibers could be formed and developed as a barrier to fully block uterine cavity. Consequently, fibroblast and myoblast cells would replace the initial native cells, e.g., stromal cells, epithelial cells, immune cells, thereby compromising the structural integrity and functional capabilities of the endometrium. The damage to the endometrial layer can lead to symptoms such as dysmenorrhea, amenorrhea, menstrual irregularities, recurrent miscarriages, and infertility [51].

Accurate diagnosis methods are indispensable for detecting and estimating the prevalence of IUAs. Currently, various diagnostic modalities are employed to visualize IUAs, including hysteroscopy, hysterosalpingography, magnetic resonance imaging (MRI), and ultrasonography techniques such as contrast sonohysterography and 3D ultrasonography. Among them, hysteroscopy is considered the gold standard for detecting IUAs. Moreover, hysteroscopic adhesiolysis is widely regarded as the preferred treatment option for uterine adhesions. As a minimally invasive surgical procedure, hysteroscopic adhesiolysis involves the use of scissors to transect and remove uterine adhesions resulting from collagen fibers under the guidance of hysteroscopy. The primary objective of hysteroscopic adhesiolysis is to restore the anatomical integrity and morphology of the uterine cavity, thereby enhancing fertility outcomes [52]. According to a ten-year (2003–2013) centralized study of hysteroscopic adhesiolysis for IUAs, 80 % of patients (a total of 638 cases) could restore a normal uterine cavity after the first hysteroscopic adhesiolysis treatment. Moreover, 95 % of women could restore a healthy uterine cavity after 3 attempts of surgery [53]. Another analysis in which a total of 355 patients suffered from IUAs were collected during January 2016 to March 2019, showing that the mean number of hysteroscopic adhesiolysis procedures were quite different due to the varying severity levels of IUAs. In order to achieve the best therapeutic effect, 1.5 attempts performed for mild IUAs, 2.8 attempts performed for moderate IUAs, and 3.3 attempts performed for severe IUAs [54].

The restoration of normal uterine cavity shape is an important sign for the improvement of fertility. The hysteroscopic adhesiolysis operation has significantly enhanced the rates of pregnancy and live birth. Clinical trials showed that the pregnancy rates increased from 32.1 % to 79.0 % and live birth rate increased from 27.2 % to 85.6 % post-operation [[55], [56], [57], [58], [59]]. Despite the great potential in fertility restoration, there still exists clinical arguments. Some researchers and surgeons claimed that alternative therapies should be considered to assist hysteroscopic adhesiolysis for IUAs treatment, especially for those suffering from severe IUAs. They indicated the recurrence of adhesions in the uterine cavity was a major complication. Clinical data demonstrated that the average recurrence rate of adhesion was approximately 20 %; however, it escalated to 62.5 % among patients with severe IUAs [60]. The high recurrence rate is a major concern. Therefore, assisted treatments, such as the utilization of intrauterine devices (IUDs) and hormone therapy, are employed to mitigate the recurrence of adhesions.

IUDs are commonly employed as adjunctive therapy for IUAs following hysteroscopic adhesiolysis, owing to their ability to act as physical barriers that effectively separate adjacent uterine walls and thereby inhibit the recurrence of adhesions [61]. IUDs are beneficial for patients with IUAs, regardless of the stage of adhesions. To achieve the best results, IUDs should be combined with other treatments, especially in patients with moderate to severe IUA. The commonly used IUDs in clinical settings encompass the Foley catheter, intrauterine balloon, and hyaluronic acid (HA) gel. Shi *et al.* conducted a single-blind randomized controlled trial involving 200 patients to assess the efficacy of intermittent intrauterine balloon therapy in preventing adhesion reformation [62]. After an 8-week follow-up period, the recurrence rates of adhesions in intrauterine balloons were significantly lower compared to the untreated groups (20.2 % versus 40.2 %). Additionally, there was a significant increase in menstrual flow. The results of the meta-analysis suggested that the utilization of IUDs for preventing adhesion reformation was highly beneficial. However, the impact of IUDs on enhancing pregnancy rates remains inconclusive. A prospective, randomized clinical trial involving 71 cases indicated that the insertion of IUDs after adhesiolysis did not yield a significant increase in patients' spontaneous pregnancy rates [63], while another comparative study showed that the ongoing pregnancy rates after IUD implantation could be up to 27 % [64].

Although Foley balloons have demonstrated significant efficacy in suppressing the recurrence rates of IUAs and improving menstrual blood flow, a notable limitation lies in their inadequate conformation to the human uterine cavity structure. The balloon's shape is inconsistent with that of the uterine cavity, thereby preventing it from accurately isolating the two sides of the uterine cavity and the uterine horns. Additionally, the localized pressure may compromise the endometrial blood supply. Recently, an innovative "heart" shaped intrauterine balloon has been developed to effectively prevent reformation of adhesions [65]. A comparative, retrospective study conducted on 150 patients with moderate or severe IUAs compared the efficacy of Foley balloon and “heart” shape intrauterine balloon for prevention of IUAs recurrence. The adhesion reformation rates rarely differed among moderate IUAs, but the “heart” shape intrauterine balloon could significantly decrease the recurrence rates among patients with severe IUAs [66].

In addition to clinical catheters and balloons, HA gel has emerged as a promising biological barrier for the prevention of adhesion recurrence. In contrast to clinically utilized catheters and balloons, HA serves as an essential endogenous component of the extracellular matrix (ECM), providing not only mechanical and structural support for separating injured endometrium but also playing a crucial role in alleviating recurrent adhesion through modulation of inflammation response and promotion of vascular effects [[67], [68], [69]]. A previous meta-analysis of randomized clinical trials participated in a total of 564 patients suffering from different severity of IUAs demonstrated the safety and efficacy of HA gel on preventing IUAs formation [58]. Acunzo [70] and Guida *et al.* [71] also proved the significant effect of HA gel on reducing the incidence and severity of IUAs after hysteroscopic surgery. However, there is currently insufficient empirical evidence to establish the superiority of HA gel over catheters and balloons in terms of efficacy. Lin *et al.* conducted a retrospective cohort study of 107 patients, suggesting IUDs and balloons were more effective than HA gel in the prevention of IUAs recurrence [72]. While Wang *et al.* performed a randomized clinical trial of 89 women indicating HA gel was advantageous in reducing IUAs reformation and increasing pregnancy rates in comparison to IUDs [73]. Additionally, Unanyan *et al.* conducted a review comparing the effectiveness of HA gel and IUDs in preventing adhesions following intrauterine surgery [74]. Their findings suggested that HA gel represented a more effective and safe approach for mitigating the recurrence of IUAs. However, Trinh *et al.* demonstrated that the combined use of HA gel and IUD could effectively prevent recurrent IUAs and reduce post-treatment American Fertility Society scores in infertile women undergoing hysteroscopic adhesiolysis [75]. More solid clinical evidence should be performed to compare the effectiveness between HA gel and conventional IUDs on the prevention of IUAs reformation. Overall, compared to other IUDs, the application of HA gel may exhibit efficacy in preventing the occurrence of IUAs, particularly in patients with mild or moderate adhesions. However, it is important to note that HA gel alone may not be sufficient for treating severe IUAs, and its use as a standalone treatment does not significantly improve pregnancy rates [76,77]. Additionally, the evidence supporting the effectiveness of HA gels in improving reproductive outcomes is currently limited and of relatively low quality. On the other hand, due to its high biocompatibility and biodegradability, HA gel can serve as a promising tissue engineering scaffold for the controlled delivery of cells/biomolecules, thereby offering potential strategies to prevent IUAs and promote uterine regeneration. A detailed discussion on this topic will be provided in Section 4.2.

On the other hand, uterine fibroids are the most common gynecologic tumors encountered by women aged 30 and above. Despite their benign nature, leiomyomas exert a profound impact on patients' quality of life. Similar with IUAs, systemic leiomyomas could also cause menorrhagia, dysmenorrhea, spontaneous abortion and infertility [78]. The clonal proliferation of smooth muscle cells is widely recognized as the primary etiology underlying leiomyomas. In clinical practice, the diagnosis of leiomyomas can be effectively accomplished through medical imaging techniques, including ultrasound and magnetic resonance imaging (MRI) in conjunction with computed tomography and hysterosonography. The surgical management of leiomyomas can be categorized into two approaches: total uterine resections, such as hysterectomy, and uterus-preserving options including myomectomy and myolysis, based on the severity of symptoms and the desire for future fertility [79,80]. Irrespective of the chosen surgical options, it is inevitable that damage to the uterus and impairment of fertility will occur. Consequently, the implementation of tissue engineering strategies becomes imperative for constructing either partial or complete bioengineered uteri to restore fertility.

## 4 Tissue engineering strategies for uterine regeneration

Cells (especially stem cells), biomolecules (e.g., growth factors, cytokines, chemokines, drugs), scaffolds (composed of natural or synthetic biomaterials) have been recognized as three vital factors in tissue engineering [43,81,82]. Accordingly, there are three main tissue engineering strategies for tissue repair and regeneration: cell-based strategies, biomolecule-based strategies, and biomaterial and scaffold-based strategies. In tissue engineering, scaffolds with interconnected pores are typically fabricated using biomaterials to facilitate the transport of oxygen and nutrients, removal of waste products, and provide mechanical support for cell growth [83,84]. Stem cells adhere to these scaffolds and subsequently infiltrate into their inner structure to undergo proliferation and differentiation into desired cell lineages for the fabrication of pre-designed tissues or organs [85]. Biomolecules are considered as regulators that modulate various cellular behaviors such as migration, invasion, proliferation, and induction of directed differentiation [86].

### 4.1 Cell-based strategies

Stem cells refer to undifferentiated cells and have the potential to self-renewal and differentiate into specific cells. Due to the regenerative potential and ability to differentiate into multiple lineages, cell-based therapy has emerged as a promising approach for the treatment of injured, damaged, and degenerative tissues [87]. At present, cell-based strategies have been extensively applied for tissue engineering and regenerative medicine, including skin [88,89], bone [90,91], neuron [92,93], muscle [94,95], etc. Meanwhile, cell-based tissue engineering strategies have been proposed as the predominant and efficacious approach for uterine regeneration, particularly in cases of impaired and thin endometrium [[96], [97], [98], [99]]. Many sources of stromal/stem cells have been proposed as efficacious for uterine regeneration, including bone marrow derived mesenchymal stem cells (BMSCs), menstrual blood derived mesenchymal stem cells (MenSCs), umbilical cord derived mesenchymal stem cells (UC-MSCs), adipose derived mesenchymal stem cells (ADSCs), endometrium mesenchymal stem cells (EMSCs), human embryonic stem cells (HESCs), and other cells, including human endometrium perivascular cells (HEPCs), human amniotic epithelial cells (HAPCs), human amniotic mesenchymal stem cells (HAMSCs) and oral mucosal epithelial cells (OMPCs). As shown in Table 1, it concludes the sources and properties of cells currently used for uterine regeneration.

**Table 1:** Sources and functions of stem/stromal cells currently used for uterine tissue regeneration.

Cell	Major Source	Function	Ref
BMSCs	Bone marrow	Upregulating estrogen and progesterone expression,	[[100], [101], [102], [103], [104]]
		Downregulating ΔNp63 level to activate epithelial related genes expression,	
		Increasing angiogenic growth factor expression, including TGF-β1, VEGF, and bFGF.	
MenSCs	Menstrual blood	Increasing angiogenesis via MAPK/AKT pathway activation and angiogenic growth factor expression,	[[105], [106], [107], [108]]
		Decreasing proinflammation response via IL-6 and IL-8 downregulation and IL1-β upregulation,	
		Higher proliferation ability than BMSCs.	
UC-MSCs	Umbilical cord	Accelerating re-epithelization and angiogenesis process,	[[109], [110], [111], [112], [113]]
		Enhancing the expression of estrogen receptor α (ERα) and progesterone receptor (PR),	
		Secreting MMP-9 to promote endometrium regeneration and restore fertility.	
ADSCs	Adipose tissue	Elevating estrogen receptors (ERα and ERβ) and progesterone receptor expression,	[[114], [115], [116]]
		Promoting angiogenic factors expression including VEGF, PDGF-BB and bFGF,	
		Suppressing inflammation response and improving vascularization,	
		Differentiating into endometrium stromal and epithelial cells.	
EMSCs	Endometrium	Inducing microvasculature formation via AKT and ERK pathways,	[117,118]
		Possessing immunosuppression ability.	
HAMSCs	Amniotic membrane	Modulating immune response via proinflammation cytokines reduction and anti-inflammation cytokines promotion,	[[119], [120], [121], [122], [123]]
		Differentiating into endometrial epithelial cells to promote endometrium regeneration,	
		Promoting angiogenesis and estrogen and progesterone receptor expression,	
HESCs	Embryo	Differentiating into endometrium-like cell,	[124,125]
		Promoting epithelization and angiogenesis.	
HEPCs	Endometrium	Inducing angiogenesis.	[126]
HAPCs	Amniotic membrane	Improving angiogenesis and stromal cell proliferation,	[[127], [128], [129]]
		Regulating the inflammatory reaction,	
		Activating autophagy via paracrine effect.	
OMPCs	Oral mucosa	Promoting re-epithelization and vascularization.	[130]

#### 4.1.1 Use of mesenchymal stem cells

The safety and efficacy of mesenchymal stem cells, including BMSCs, MenSCs, UC-MSCs, ADSCs, EMSCs, and HAMSCs in promoting tissue regeneration have been demonstrated through a range of preclinical and clinical trials [99]. Given the intricate and dynamic microenvironment within the human uterus, conventional therapies are insufficient in facilitating uterine regeneration and restoring fertility. Therefore, cell-based tissue engineering strategies utilizing mesenchymal stem cells is favored for treating uterine tissue defects and restoring fertility.

BMSCs derived from bone marrow are a type of adult stem cells possessing various crucial characteristics and capabilities, including self-renewal, multi-lineage differentiation potential, and reduced immunogenicity. A previous study postulated that endometrial stem cells might exhibit comparable therapeutic effects to BMSCs [131]. The hypothesis aligned with the dual functionality of BMSCs in uterine regeneration: 1) direct differentiation into endometrial epithelial cells to enhance gland and blood vessel formation, and 2) stimulation and activation of resident endometrial stem cells through paracrine signaling. Consequently, a tissue engineering approach based on BMSCs, either via direct injection or seeding on/in 3D scaffolds, has been recognized as a potent therapeutic strategy for uterine regeneration [103,104,132].

BMSCs were the pioneering stem cells to be clinically employed for treating patients with severe IUAs in 2011 [133]. As a result, the findings demonstrated that direct uterine injection of BMSCs significantly enhanced pregnancy rates. Subsequently, there has been a remarkable surge in research and application of BMSCs for uterine regeneration. For example, the transplantation of BMSCs via vein or intrauterine injection could enhance endometrium thickness and promote pregnant rates as well as embryo numbers in rat IUAs models [134,135]. In addition, the clinical studies demonstrated that intrauterine injection of autologous BMSCs could significantly improve uterine cavity with enhanced endometrium thickness and intensity of menstrual blood in the refractory IUA patients [136]. But the problems are inevitable. Due to the dynamic and complex nature of the intrauterine environment, which is also acidic, it is not conducive for the survival and growth of BMSCs. Consequently, direct intrauterine transplantation of BMSCs may trigger cell death and apoptosis, leading to suboptimal therapeutic outcomes. Conversely, intravenous injection of BMSCs would subject them to systemic circulation, potentially resulting in limited bioavailability and efficacy. Consequently, only a minimal number of BMSCs could ultimately reach their intended intrauterine targets. For example, a recent study indicated that the survival rate of intravenous injection of BMSCs was 0.264 % and 0.217 % after 2- and 3-week implantation [137]. Therefore, achieving a desirable therapeutic effect necessitates a high cell number and concentration injection due to the limited bioavailability of the cells.

In order to address the limitations of direct implantation of BMSCs, 3D scaffolds have been employed as carriers or vehicles for BMSCs to enhance cell viability and reduce apoptosis. Compared to intrauterine and intravenous cell injection, scaffolds composed of hyaluronic acid, collagen, and/or other biomaterials can offer mechanical support and provide attachment sites for BMSCs. Moreover, the hierarchical and porous structure of these scaffolds facilitates oxygen and nutrient transport, thereby promoting cell survival and growth. Yang *et al.* proposed a viable strategy for enhancing endometrium regeneration by combining Pluronic F-127 (PF-127) hydrogel with BMSCs [138]. The PF-127 hydrogel could promote the survival and viability of BMSCs *in vitro* and improve angiogenesis and inhibit proinflammatory effect *in vivo*. After implantation, the endometrium thickness and gland number were increased while the fibrosis area was decreased. Currently, collagen scaffolds have emerged as the predominant substrates for loading and encapsulating stem cells and/or biomolecules to facilitate uterine regeneration. Notably, since the early 21st century, collagen scaffolds loaded with BMSCs have been extensively employed for effectively regenerating severe uterine injuries. As shown in Fig. 3A–D, the transplantation of collagen/BMSCs composite scaffolds could increase endometrium thickness and facilitate microvasculature reconstruction, thereby facilitating the regeneration of uterine structure and functions [139]. To further enhance the regenerative capacity, basic fibroblast growth factor (bFGF)-transfected BMSCs were loaded on collagen scaffolds for repairing uterine tissue defects. As a result, collagen/bFGF-BMSCs could significantly promote angiogenesis and microvascular density and therefore facilitate uterine tissue regeneration (Fig. 3E–G). [132]. Additionally, a randomized, controlled clinical trial involving 140 participants revealed that autologous BMSCs-scaffold transplantation could increase the endometrium thickness and thus increase patients’ ongoing pregnancy and live birth rates [140].

The safety and efficacy of BMSCs for endometrium regeneration and restoration of uterine structure and functions showed promising results, whether administered through intravenous or intrauterine injection, or by seeding BMSCs on scaffolds. However, the underlying mechanisms governing uterine regeneration by BMSCs remain poorly elucidated. The expression of estrogen (ER) and progesterone (PR) is pivotal for reproductive activity [141]. ER and PR serve as essential mediators to activate downstream gene expression and modulate cell differentiation, thereby maintaining uterine functions. However, in damaged endometrium, the expression of ER and PR fails to be maintained at a normal level. Wang *et al.* proposed that either intrauterine transplantation or intravenous injection of BMSCs could elevate gland number and increase endometrium thickness by upregulating ER and PR expression [102]. They emphasized the crucial role of ER and PR as transduction signals for uterine regeneration, suggesting that BMSCs administration could facilitate their expression and further promote tissue regeneration. Another clinical trial indicated that BMSCs could downregulate ΔNp63 expression and therefore normalize the stemness alteration and recover the structure and functions of uterine tissues [101]. ΔNp63, a member of the p53 family, is an important factor to modulate the balance between stemness and differentiation in epithelial development. The expression of ΔNp63 in patients suffering from endometrium injury could be more than 100-fold higher than the normal level. The overexpression of ΔNp63 would result in endometrial quiescence, causing a reduction in the expression of epithelial-related genes. Simultaneously, estradiol failed to stimulate the epithelial cells when there was an ectopic overexpression level of ΔNp63. The clinical trials showed that the BMSCs transplantation could activate ΔNp63-induced endometrial quiescence and subsequently reconstruct endometrium and restore fertility. Additionally, the stromal cell-derived factor-1 (SDF-1) and C-X-C chemokine receptor type 4 (CXCR4) signaling pathway have been considered as a potential mediator in the implantation of BMSCs [142]. After uterine tissue injury, the upregulation of SDF-1 facilitated the engraftment and directed migration of BMSCs towards the injured sites, thereby promoting self-regeneration. Moreover, BMSCs exhibited a paracrine effect after implantation by enhancing angiogenic growth factor expression and modulating the inflammatory process, ultimately facilitating uterine regeneration [100].

**Fig. 4:**
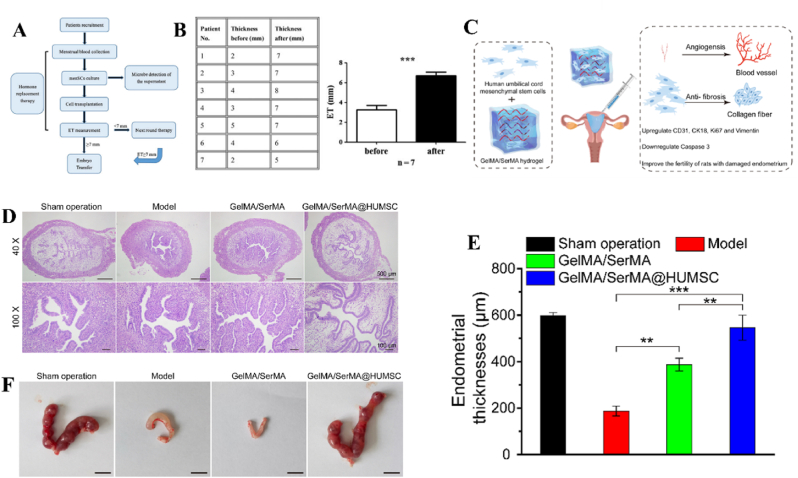
MenSCs transplantation for uterine injury treatment. (A) The clinical procedure of MenSCs injection for IUAs patients. ET: endometrium thickness. (B) The patients' endometrium thickness before and after MenSCs treatment [145]. An injectable hydrogel loaded with UC-MSCs for the treatment of uterine injury. (C) Hydrogel encapsulating UC-MSCs for the treatment of uterine injury by injection. (D) H&E staining images showing the morphology of uterine tissues after treatment. (E) Endometrium thickness and (F) embryo numbers in rat uterus after treated by hydrogels loaded with UC-MSCs [146].

In addition to BMSCs, other mesenchymal stem cells, including MenSCs, UC-MSCs, ADSC, EMSCs, and HAMSCs have emerged as prominent candidates in preclinical and clinical trials for uterine defect repair. One major concern for BMSCs implantation is the cell source. Primarily, the extraction of autologous BMSCs may potentially induce additional harm to the patient, while transplantation of allogeneic BMSCs raises ethical concerns and poses risks of immune rejection. In comparison to BMSCs, MenSCs derived from menstrual blood exhibit enhanced self-renewal capabilities and can be easily obtained through a non-invasive procedure directly from patients themselves, thereby eliminating ethical concerns and minimizing the risk of immune rejection. Apart from the fundamental properties of MSCs, MenSCs perform higher extraction efficiency and longer passaging capacity [143]. Another advantage of MenSCs is their strong homing ability to injured tissue, suggesting that they can be easily chemoattracted by SDF-1. Moreover, MenSCs demonstrate superior proliferation capacity compared to MSCs derived from bone marrow, adipose tissue, umbilical cord, etc. Several studies have revealed that MenSCs exhibited a comparable therapeutic efficacy to BMSCs in promoting uterine regeneration. Likewise, MenSCs also exhibit the ability to promote endometrium thickness, gland number, epithelization, angiogenesis as well as decrease proinflammation [108,144]. The *in vivo* studies demonstrated that the therapeutic effect of MenSCs on uterine tissue reconstruction mainly resulted from their ability to enhance angiogenesis and suppress proinflammatory responses. The enhanced angiogenic effect on endometrium regeneration was mainly due to the activation of p38 mitogen-activated protein kinase (MAPK) and protein kinase B (AKT) pathway [105,107]. And the suppressed proinflammatory responses was fundamentally ascribed to the downregulation of crucial inflammatory cytokines, such as interleukin-6 (IL-6) and interleukin-8 (IL-8) and the reduction of pro-inflammatory cytokines, IL1-β. For example, Zhang *et al.* indicated that the implantation of MenSCs could greatly improve endometrium proliferation and angiogenesis while concurrently reducing inflammation [106]. Besides, a non-controlled and prospective 3-year clinical study involving seven patients with severe IUAs proved the therapeutic efficacy of autologous MenSCs implantation treatment [145]. Following multiple rounds of MenSCs therapy combined with hormone treatment, the therapeutic outcomes revealed that the endometrial thickness of five patients increased to 7 mm (Fig. 4A and B). Encouragingly, two patients achieved conception after undergoing frozen embryo transfer. Notably, one patient experienced spontaneous pregnancy following a second MenSCs transplantation.

The application of UC-MSCs for tissue regeneration has been extensively investigated in preclinical and clinical studies due to their easy collection, low immunogenicity, and robust proliferative capacity [147]. Although UC-MSCs have been predominantly used for repairing bone [148,149], cartilage [150,151], skin [152,153], there are still a few cases for endometrium reconstruction [109,146]. The direct injection of UC-MSCs via rat tail vein was carried out to explore their therapeutic safety and efficacy in endometrium regeneration. As a result, UC-MSCs could reduce the expression of α-smooth muscle actin (α-SMA) and transforming growth factor-β (TGF-β) and concurrently increase reepithelization through upregulating the expression of endometrial stromal marker vimentin and epithelial marker cytokeratin-19 (CK-19). The expression of angiogenic factors such as CD31 and vascular endothelial growth factor-A (VEGF-A), were also enhanced. Additionally, the associated inflammatory factors were modulated through the downregulation of tumor necrosis factor-α (TNF-α) and IL-2 ^110^. Collagen scaffolds have gained significant popularity as promising carriers for the delivery of UC-MSCs to facilitate the restoration and regeneration of uterine structure and functions. Xin *et al.* fabricated collagen scaffolds loaded with human UC-MSCs to facilitate endometrium regeneration and restore fertility in a murine model [109]. The *in vivo* results indicated that UC-MSCs could induce intrinsic endometrium cell proliferation and epithelium recovery, enhance the expression of essential hormones receptors including ERα and PR through paracrine effect, thereby promoting endometrium reconstruction. Meanwhile, UC-MSCs mixed with gelatinous degradable collagen fibers were transplanted into the uterine cavity for relieving rat uterine scar. UC-MSCs secreted matrix metalloproteinase-9 (MMP-9) to facilitate collagen degradation and promote endometrium regeneration as well as improve receptive fertility restoration [111]. Recently, Chen *et al.* constructed an injectable gelatin/sericin hydrogel loaded with UC-MSCs for the treatment of uterine injury [146]. The application of hydrogels loaded with UC-MSCs has the potential to enhance endometrial thickness, thereby promoting increased receptivity for embryo transfer (Fig. 4C–F). In addition to animal models, the application of UC-MSCs in clinical trials have been further investigated. Cao *et al.* conducted a phase I clinical trial in 26 patients suffering infertility resulted from severe IUAs with 30-month follow-up [154]. They transplanted collagen scaffolds loaded with UC-MSCs into patients’ uterine cavities. Consequently, after 3-month operation, the endometrium thickness of patients increased, and the expression of estrogen receptors upregulated. By the end of the follow-up, ten patients achieved pregnancy, and among them, eight successfully delivered healthy infants, thereby substantiating the safety and efficacy of uterine regeneration using UC-MSCs.

**Fig. 5:**
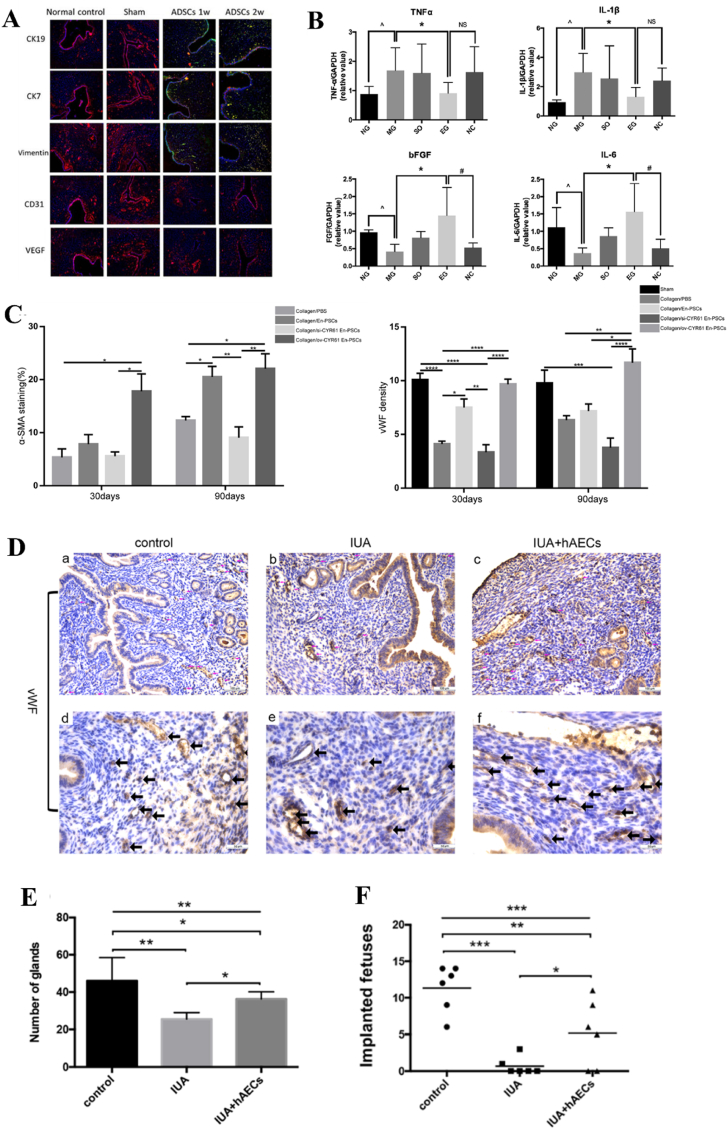
(A) Immunofluorescence images showed that ADSCs could differentiate into endometrium epithelial cells and promote angiogenesis *in vivo* [114].(B) HAMSCs could significantly reduce the expression of proinflammatory cytokines (TNF-α and IL-1β) and increase anti-inflammation cytokines (bFGF and IL-6) expression after transplantation [119]. (NG: normal group, SO: shame-operated group, MG: mechanical injury group, EG: HAMSCs transplantation group, NC: negative control group). (C) The statistical analysis of the α-SMA expression and capillary vessels number after CYR61 genes-transfected HEPCs treatment [126]. (D) Immunofluorescence staining of vWF showing the micro-vessels density in the endometrium. (E) The number of glands and (F) implanted fetus after HAECs treatment [127].

BMSCs, MenSCs, and UC-MSCs have already been employed in clinical practice for uterine regeneration, while other stem cells such as ADSCs, EMSCs, and HAMSCs remain at the preclinical stage of investigation. Their safety and effectiveness, however, have been convincingly demonstrated in preclinical trials; nevertheless, further endeavors are needed to explore their potential for clinical application. Sun *et al.* conducted a novel approach for uterine remodeling via cell sheet engineering. They engineered ADSCs to form scaffold-free cell sheets *in vitro* and then injected them into rat uterine cavities. The ADSCs cell sheet not only exhibited differentiation potential towards stromal cells, but also exerted an autocrine effect to enhance endometrium and myometrium proliferation, as well as induce angiogenesis [155]. Additionally, Shao *et al.* indicated that the injection of ADSCs into the uterine cavity resulted in an upregulation of both estrogen receptor (both ERα and ERβ) and PR, exhibiting similar behavior to BMSCs [114]. The *in vivo* results showed that ADSCs could differentiate into endometrium epithelial cells, thus improving endometrial injury and promoting pregnancy rate (Fig. 5A). Another comparative investigation revealed that ADSCs performed a superior capacity to promote angiogenesis for endometrium regeneration compared to UC-MSCs and EMSCs [115]. The expression of proangiogenic factors including VEGF, platelet derived growth factor-BB (PDGF-BB) and bFGF, had the highest level after ADSCs treatment.

The safety and efficacy of EMSCs for the repair of uterine injury have also been explored. The underlying mechanisms responsible for uterine regeneration by EMSCs closely resemble those observed with BMSCs. EMSCs facilitated restoration of uterine structure and functions through induction of angiogenesis, potentially mediated by AKT and ERK signaling pathways [156]. EMSCs play an essential role in endometrium thickness. The thickness of endometrium can grow from 0.5-1 mm to 5–7 mm within a week due to the self-renewal and proliferative properties of EMSCs [157]. Gan *et al.* initially investigated the function of HAMSCs for uterine regeneration and fertility restoration [119]. Compared to the aforementioned cells, HAMSCs are a readily available, abundant, immunoprivileged cell source. The regeneration capacity of HAMSCs is probably due to immunomodulatory properties. After implantation, HAMSCs could significantly reduce the expression of proinflammatory cytokines such as TNF-α and IL-1β and increase anti-inflammation cytokines expression (bFGF and IL-6) (Fig. 5B). HESCs are a promising candidate for cell-based therapy because of its pluripotent and highly proliferative properties. Previously, HESCs loaded on collagen scaffolds were transplanted into the uterine cavity for endometrium regeneration [124]. HESCs could differentiate into endometrium-like cells and promote epithelialization *in vivo*.

#### 4.1.2 Use of other cells

In addition to mesenchymal stem cells, other stem cells or somatic cells were attempted to treat uterine defects and restore fertility. HEPCs performed like endometrium stem cells in the endometrium. Perivascular cells exhibit myogenic potential and migration ability. Moreover, they present multiple potency, and they can differentiate into chondrocytes, adipocytes, phagocytes, osteoblasts, and granulocytes. For instance, Li *et al.* fabricated collagen scaffolds loaded with HEPCs to promote angiogenesis for the repair of a full-thickness uterine injure in a rat model [126]. In this study, HEPCs were transfected with cysteine-rich angiogenic inducer 61 (CYR61) overexpression plasmid. The upregulation of CYR61 expression in HEPCs facilitated angiogenesis and vascularization, leading to enhanced regeneration of the endometrium and myometrium, thereby improving the pregnancy rate (Fig. 5C).

HAECs derived from human placenta possess the potential to serve as alternative stem cells for tissue engineering and regenerative medicine due to their remarkable differentiation multipotency, low immunogenicity, low tumorigenicity, and abundant sources. Some preclinical and clinical investigations have substantiated the safety and efficacy of HAECs in regenerating diverse tissues, including bone, neuron, cartilage, skin, etc., [158]. However, the function of HAECs in facilitating endometrium regeneration and restoring fertility has been rarely conducted. Recently, the therapeutic potential of HAECs was investigated by implanting HAECs into a murine IUA model via intraperitoneal injection [127]. The administration of HAECs significantly enhanced endometrial thickness, glandular density, microvasculature formation, and stromal cell proliferation following an 8-day surgical procedure, ultimately leading to increased pregnancy rates (Fig. 5D–F). The promotion effect on injured endometrium may be attributed to the activation of autophagy pathway through paracrine signaling. OMECs can be harvested and obtained by minimally invasive operation and perform high proliferate capability. The abundant sources and extensive proliferation potential render OMECs a compelling cellular alternative for tissue regeneration. Kuramoto *et al.* found that the injection of OMECs into the uterine cavity could inhibit the formation of fibrosis area [159]. However, direct implantation of OMECs into the uterine cavity would compromise cell viability and induce apoptosis. Therefore, Chen *et al.* seeded OMECs on decellularized and lyophilized amniotic membranes and then implanted the scaffolds into the uterine cavity for regenerating injured endometrium [130]. After treatment, the uterine cavity structure was recovered, the epithelium and endometrium glands were increased, and the microvasculature density and expression of VEGF were promoted.

### 4.2 Biomolecule-based strategies

Biomolecules, including growth factors, cytokines, chemokines, hormones, etc., play a vital role in modulating cell growth and differentiation, thereby expediting the tissue regeneration cascade [[160], [161], [162]]. Many biomolecules have been employed in preclinical and clinical trials to treat defective and degenerative diseases. Likewise, biomolecule-based tissue engineering strategies applying various biomolecules to promote endometrium regeneration and reconstruct uterine structure holds great promise for the treatment of uterus defects and restoration of fertility. As shown in Table 2, it presents the mostly employed biomolecules for uterine regeneration.

**Table 2:** Biomolecules used in uterine tissue regeneration.

Biomolecule	Function	Ref
SDF-1α	Recruiting and homing stem cells to the injured site.	[142,[163], [164], [165]]
17β-estradiol/estradiol valerate	Augmenting the effect of angiogenic growth factors, Modulating inflammatory process via steroid metabolism enzymes and receptors and innate immune cells.	[[166], [167], [168]]
VC	Regulating inflammation response and reducing apoptosis.	[138,169,170]
bFGF	Promoting angiogenesis and inhibiting inflammation effect.	[132,[171], [172], [173]]
VEGF	Promoting endothelial cells proliferation and migration, and blood vessels reconstruction.	[100,[174], [175], [176]]
LIF	Downregulating pro-inflammation cytokines and upregulating anti-inflammation cytokines.	[177,178]
PRP	Co-culturing with stem cells to reduce cell apoptosis and improve cell growth, Regulating cell behaviors and promoting damaged uterine healing via paracrine effect.	[106,[179], [180], [181], [182], [183], [184]]

#### 4.2.1 SDF-1α

SDF-1α, also known as C-X-C motif chemokine 12 (CXCL12), is a widely recognized chemokine protein that exhibits remarkable ability in adhering to, homing to, and recruiting stem cells in tissue engineering and regenerative medicine [[185], [186], [187]]. SDF-1α and its exclusive receptor, CXCR4, are expressed in many stem cells such as hematopoietic stem cells (HSCs) and mesenchymal stem cells. Injured tissues can secrete a substantial amount of SDF-1α, leading to an increased localized concentration of SDF-1α, which subsequently guides stem cells to defective sites through the SDF-1α/CXCR4 axis. Consequently, in-situ delivery of SDF-1α could effectively recruit stem cells and facilitate their migration towards the injured site, thereby expediting the process of tissue regeneration. The efficacy of SDF-1α has been confirmed both *in vitro* and *in vivo*, establishing it as a promising biomolecule for the treatment of tissue defects [[188], [189], [190]].

**Fig. 6:**
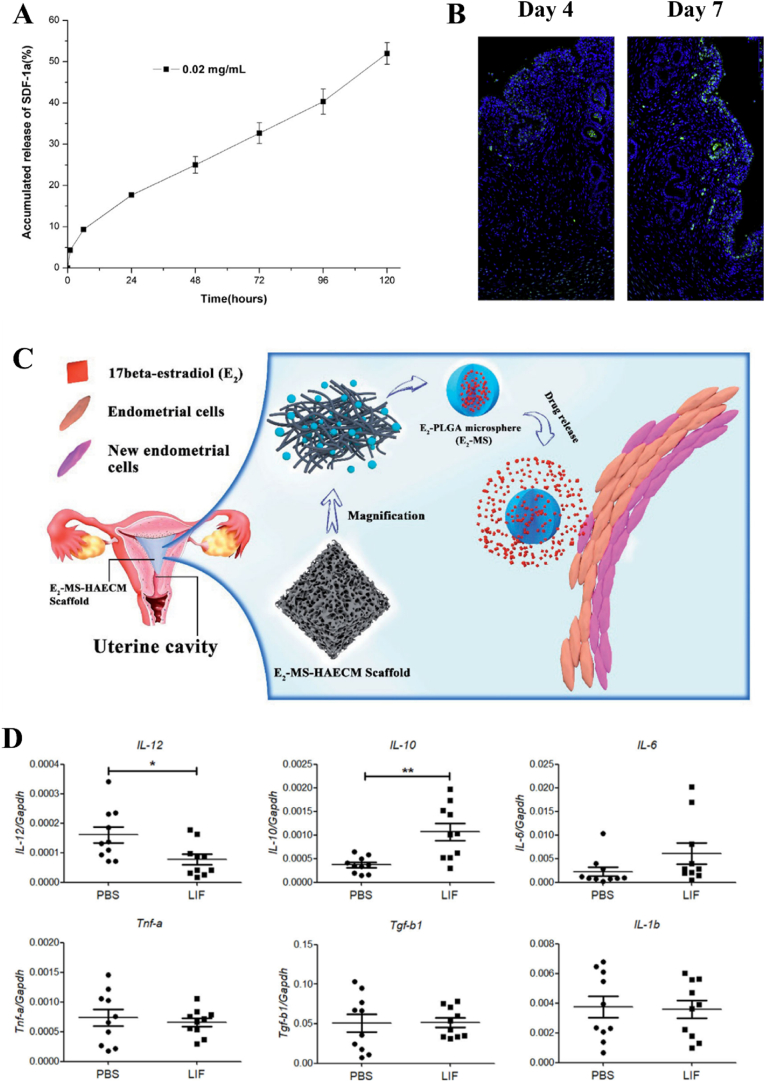
The functions of biomolecules on uterine regeneration. (A) The sustainable SDF-1α release behavior of chitosan-heparin hydrogels. (B) The fluorescence images of c-kit positive cells at the injured site (Blue: nuclei, Green: c-kit) when treated with SDF-1α-loading hydrogels [163]. (C) Schematic illustration for scaffolds loaded with 17β-estradiol for uterine regeneration [167]. (D) mRNA expression level of inflammatory cytokines in injured site after treated with collagen/LIF scaffolds for 1 week. ∗p < 0.05; ∗∗p < 0.01 ^177^. (For interpretation of the references to colour in this figure legend, the reader is referred to the Web version of this article.)

Because most of uterine-related diseases such as thin endometrium thickness and IUAs usually result from endometrium stem cells deficiency and low cell viability, the application of SDF-1α, especially those in-situ biomolecule release, to recruit stem cells into endometrium site exhibits great potential for endometrium regeneration. Ersoy *et al.* demonstrated that SDF-1α could promote stem cell recruitment and uterine repair in a mice IUAs model [142]. In their study, SDF-1α solution (10 mg/kg/day) and bone marrow derived stem cells solution were directly injected into the uterine. SDF-1α increased bone marrow derived stem cells engraftment and recruitment via SDF-1α/CXCR4 axis. As a result, the fertility rate and litter size were increased. However, the challenge lies in the fact that direct intrauterine injection of SDF-1α fails to exhibit sustained drug release behavior and consequently lacks the ability to prolong therapeutic effects, thereby indicating its limited efficacy in treating severe uterine diseases. The application of hydrogels as a promising candidate for the encapsulation and controlled release of biomolecules in physiological environments is highly advantageous for tissue regeneration [[191], [192], [193]]. The use of hydrogels as SDF-1α vehicles can not only preserve its bioactivity but also enable a controlled release behavior, thereby achieving a desirable therapeutic outcome. As mentioned, chitosan-heparin hydrogels were employed to release SDF-1α in-situ for damaged endometrium healing in a rat model (Fig. 6A and B) [163]. The increased migration of endogenous c-kit positive hematopoietic stem cells (HSCs) in the SDF-1α group can be attributed to the recruitment behavior of SDF-1α towards these stem cells. Meanwhile, compared to one-off SDF-1α injection, hydrogels loaded with SDF-1α performed long-term HSCs recruitment and demonstrated superior therapeutic efficacy. The more HSCs migrated to the endometrium injury site, the more endogenous cytokines including VEGF and TGF-β1 would be secreted and involved in injury repair, thereby resulting in the promotion of uterine regeneration. Another reported scaffold used for SDF-1α encapsulation was silk-cellulose hydrogels [164]. Cai *et al.* suggested that SDF-1α was more efficacious than other reported growth factors such as VEGF and bFGF because SDF-1α as a homing factor could promote uterine cells migration *in vitro* and facilitate vascularization and epithelization through recruitment of endothelial cells *in vivo*. Previous studies have demonstrated that other growth factors, such as VEGF and bFGF, were found to facilitate blastocyst implantation approximately 90 days post-surgery. In contrast, SDF-1α exhibited the potential to enhance endometrium regeneration and improve pregnancy outcomes within a shorter timeframe of 30 days post-surgery for repairing full-thickness uterine injuries.

#### 4.2.2 Hormones

The use of hormone therapy, specifically estrogen and progestogen, as a means to facilitate endometrium regeneration is a prevalent therapeutic approach in clinical practice. Generally, hormone therapy serves as a complementary method to suppress fibrosis formation and promote the reconstruction of injured endometrium subsequent to hysteroscopic hydrolysis or to augment the functions of other treatments such as IUDs and intrauterine scaffolds. Plenty of preclinical and clinical trials have affirmed the safety and efficacy of hormone therapy for the treatment of uterine tissue defects [[194], [195], [196]].

Estrogen plays a vital role in improving endometrium function layer growth and reconstruction after menstruation. There are plentiful estrogen receptors in endometrium stromal cells and glandular epithelial cells. When estrogen binds to its receptors, it would exert its main function to augment the effect of angiogenic growth factors such as VEGF, bFGF and TGF-β1. Afterward, these growth factors would promote migration, proliferation, differentiation, and tube formation of endothelial cells, thereby augmenting micro-vessel density and promoting angiogenesis [197]. Many studies have demonstrated the promotion effect of estrogen therapy on VEGF expression, glandular epithelium area, and blood vessels density [198,199]. On the other hand, estrogen and progesterone could regulate the inflammation process in human endometrium. The modulation of inflammatory response encompassed a complex interplay of inflammatory mediators, including steroid metabolism enzymes and receptors, as well as innate immune cells [200].

In clinic, estrogen as a postoperative treatment is recommended to be orally administered. However, the lack of standardized specifications regarding dosage, efficacy, and route of administration remains evident due to limited and inconsistent data collected from various trials. According to the recommendation of AAGL Practice Guideline, hormone therapy is deemed necessary for facilitating endometrium regeneration; however, there remains uncertainty regarding the optimal dosage of estrogen [201]. Consequently, a lack of consensus among surgeons persists regarding both the dose and cycle of estrogen therapy for preventing recurrent IUAs and promoting endometrial thickness. Current clinical trials indicate that the prevailing hormone therapy involves a 2- to 3-month treatment combining estrogen and progesterone. However, the dosage of estrogen ranges from 0.625 mg to 6 mg estradiol valerate or 17β-estradiol, with or without supplementary administration of progesterone. Some surgeons prefer higher dose and long period hormone treatment. Some clinical studies compared the safety and therapeutic effect of different doses of estrogen therapy [202]. For example, Liu *et al.* conducted a cohort study to compare the therapeutic effect of 4 mg and 10 mg daily dose of post-surgery estradiol valerate therapy for recurrent IUAs prevention [203]. A total of 176 patients in which 91 received 10 mg daily estradiol valerate and 85 received 4 mg daily estradiol valerate were analyzed. After 21–28 days treatment, both groups improved endometrium thickness. However, there was no difference in preventing the fibrosis formation, suggesting that higher doses of estrogen therapy did not necessarily result in enhanced therapeutic efficacy. However, other clinical investigations supported that the higher dose of estrogen treatment was superior to the low dose. Liu *et al.* randomly divided 120 IUAs cases into 3 mg and 9 mg estradiol valerate treatment [204]. As a result, 9 mg estradiol valerate per day was beneficial for moderate and severe IUAs. Whereas, a recent study has reported that postoperative estrogen therapy did not demonstrate efficacy in reducing the incidence or severity of adhesion reformation, nor did it show improvement in menstrual patterns [196].

The oral administration approach for hormone therapy has intrinsic limitations. Estrogen, either estradiol valerate or 17β-estradiol, is poorly water-soluble and presents limited half-life, which can lead to insufficient bioavailability [205]. Consequently, in order to achieve a desirable therapeutic effect, it is recommended that patients administer an adequate oral dosage of estrogen. However, due to the lack of targetability in oral administration of estrogen, it may result in suboptimal drug concentration at injured endometrium sites, thus leading to wastage. To effectively utilize estrogen therapy and address these limitations, the introduction of a controlled drug delivery system such as an in-situ injectable hydrogel is necessary [206]. Feng *et al.* conducted a multicenter randomized controlled study showing that the intrauterine estrogen-releasing system was more effective at reducing adhesion than traditional oral estrogen [207]. The endometrium was thicker in the intrauterine estrogen-releasing system in comparison to traditional oral estrogen. Moreover, the rate of improvement in menstruation was greater in the intrauterine estrogen-releasing system. The Heparin-poloxamer (HP) hydrogels have been prepared as a 17β-estradiol sustained-release delivery system for endometrium regeneration [166,208]. Zhang *et al.* implied that the therapeutic effect of HP hydrogel loaded with 17β-estradiol in improving gland number and vascularization was mainly attributed to continuous release of estradiol, which could suppress ER stress signaling via the activation of PI3K/Akt and ERK1/2 pathway and upregulate kisspeptin via ERK1/2 and MAPKs p38 pathways. Moreover, Yao *et al.* fabricated an in-situ injectable 17β-estradiol-loading Aloe/poloxamer hydrogel scaffold to facilitate endometrium repair and inhibit recurrent IUAs [209]. The hydrogels with temperature sensitivity and multi-therapeutic effect would significantly improve cargo solubility and prolong estradiol release period, thereby increasing uterine morphology recovery. Furthermore, as shown in Figs. 6C and 17β-estradiol could be incorporated in poly(lactic-co-glycolic acid) (PLGA) microspheres to sustainably release the estradiol for 21 days to improve endometrium regeneration [167].

#### 4.2.3 Other biomolecules

L-ascorbic acid, also known to as Vitamin C (VC), is an essential biomolecule in mammals for the maintenance and regulation of hemostasis. It is well-known that VC is a strong reducing chemical, which can inhibit the formation of intracellular reactive oxygen species. Additionally, VC has been defined as a regulator to control the folding and deposition of collagen proteins and thus can affect the structures and properties of extracellular matrix. Recently, scientists discovered that VC could be an important chemokine to relieve inflammatory process and manipulate cell activities, such as proliferation and differentiation [169]. VC could promote uterine regeneration through enhancing collagen synthesis, reducing oxidative stress, and modulating the immune response. Due to its inherent advantages, VC has been extensively applied in tissue engineering and regenerative medicine, including uterine regeneration. Yang *et al.* used VC to assist BMSCs in Pluronic F-127 hydrogel system [138]. They found that VC could improve cell viability, such as promoting the survival and growth of BMSCs in PF-127 hydrogels and meanwhile reducing the apoptosis. Consequently, VC demonstrated its ability to protect and prolong the function of BMSC *in vivo*, thereby enhancing the reconstruction of damaged endometrium post-implantation.

bFGF is a potent growth factor to promote angiogenesis by directly affecting endothelial cells and indirectly upregulating VEGF and inhibit inflammation process in endometrium regeneration. bFGF could facilitate endometrium stromal cell proliferation and improve micro-vessel density and epithelization through autocrine interaction when binding with its receptors [210]. Moreover, bFGF could enhance extracellular matrix formation, thereby creating an optimal environment for uterine tissue regeneration. The dysfunction of endometrium leads to a reduction in bFGF levels, subsequently resulting in compromised vascularization and infertility. Considering that the endogenous secretion of bFGF during the self-healing process is insufficient for effective regeneration of the endometrium, it is imperative to develop a sustained delivery system for in-situ release of bFGF at defective endometrial sites [132]. As aforementioned, collagen scaffolds loaded with bFGF by fusing a collagen-binding domain to the N-terminal of native bFGF could effectively promote the regeneration process of severe murine uterine damage [173]. The controlled delivery of bFGF significantly improved endometrium and muscle cell proliferation. Subsequently, this controlled drug delivery system was applied in clinical practice [172]. 18 patients with scarred endometrium were treated by collagen scaffolds loaded with collagen-binding bFGF. As a result, the blood vessel density and endometrium thickness increased while the scarred endometrium area decreased. Notably, 3 patients achieved successful pregnancies.

VEGF is another crucial angiogenic factor involved in uterine regeneration. VEGF plays a crucial role in uterine tissue regeneration by promoting angiogenesis, re-epithelialization, and influencing endometrial receptivity. Previous studies have confirmed that the upregulation of VEGF exerted beneficial effects on the migration and proliferation of endothelial cells, thereby promoting blood vessel reconstruction during defects repair [211]. Due to the short half-life, dosage, and bioavailability problems, a controlled-release approach inspired by bFGF delivery system was employed. Specifically, collagen scaffolds loaded with collagen-binding VEGF were utilized to prolong its therapeutic effect and promote scarred uterus remodeling [174]. Li *et al.* further demonstrated the therapeutic efficacy of VEGF in promoting endometrium reconstruction [100]. They generated VEGF-transfected BMSCs and then transplanted cells to rats via tail vein injection. Consequently, the transplantation of VEGF-transfected BMSCs resulted in an increase in endometrial thickness and blood vessel density, restoration of uterine morphology, and improvement in fertility through the secretion of VEGF.

Leukemia inhibitory factor (LIF), an effective cytokine belonging to the IL-6 family, plays an essential role in a series process, i.e., development, hematopoiesis, inflammation, and regeneration [[212], [213], [214]]. Moreover, LIF is indispensable for uterine receptivity and blastocyst implantation. The primary site of LIF expression in the uterine is localized to the glandular and luminal epithelium. However, there is little known regarding its role in uterine regeneration and blastocyst implantation. LIF may modulate endometrial cell proliferation, differentiation, and remodeling, as well as enhance endometrial receptivity for embryo implantation through its action on immune modulation and activation of key signaling pathways. Infertility may result from the absence of LIF due to uterine endometrial damage. Recently, Xue *et al.* investigated the function of LIF on endometrium regeneration [177]. They used collagen scaffolds as vehicles to encapsulate LIF. As shown in Fig. 6D, the implantation of collagen/LIF scaffolds promoted the regeneration of rat uterine horns with full-thickness injury through the inhibition of inflammatory cells infiltration and downregulation of proinflammatory cytokine IL-12 expression and upregulation of anti-inflammation cytokine Il-10 expression. The results, though, suggested the uterine regeneration was partially due to immunomodulatory properties, the therapeutic effect of LIF on uterine regeneration should be further explored.

#### 4.2.4 Platelet-rich plasma

Platelet-rich plasma (PRP) has gained considerable attention in tissue engineering and regenerative medicine in the last three decades [[215], [216], [217]]. PRP derived from blood refers to an accumulation of platelet rich plasma protein. PRP could be defined as autologous PRP and allogeneic PRP based on different sources. Despite limited resources, autologous PRP has been extensively used in preclinic and clinic practice due to its immunologically inert nature. PRP products can be also recognized as two categories dependent on whether leukocyte is contained or not, leukocyte-rich PRP (L-PRP) and pure PRP (P-PRP) [218]. The simple preparation method that comprises centrifuging to remove the red blood cell and activation to form PRP gels makes it easy for applications. To date, considering that PRP contains various cytokines, growth factors, and nutrients, which enable to modulate cell fate and behavior(e.g., cell migration, invasion, attachment, proliferation, and differentiation), the application of PRP for tissue repair and regeneration and anti-inflammation purposes has gained a significantly surge [219,220]. For example, Zhang *et al.* indicated that human MenSCs cultured in 10 % activated PRP exhibited a higher proliferation rate and decreased apoptosis rate than those cultured in 10 % fetal bovine serum, suggesting that PRP could provide nutrient for cell growth and promote cell proliferation [106]. Tremendous growth factors and cytokines in PRP such as platelet-derived growth factor, TGF, insulin-like growth factor (IGF), VEGF, epidermal growth factor (EGF), FGF, interleukin, play a vital role in angiogenesis, osteogenesis, anti-inflammation, anabolism. These growth factors and cytokines released from PRP can affect and regulate tissue regeneration through paracrine signaling pathways.

Due to its attractive advantages, the application of PRP has been investigated in recent years for endometrium regeneration and partial defective uterine reconstruction [221,222]. As aforementioned, although cell-based therapy exhibits significant efficiency for uterine tissue engineering, the survival of implanted stem cells is a great challenge. If there are no supports protecting and assisting stem cells, stem cells would just survive for several days, which is not beneficial for tissue regeneration. Generally, scientists typically prefer to initially employ collagen scaffolds as the vehicle for seeding and culturing stem cells *in vitro* and subsequently implant stem cells-loaded collagen scaffolds into damaged uterine sites for regeneration [109,124,139,154]. In contrast, in comparison to collagen scaffolds, PRP can emerge as a more promising carrier for cell loading and growth due to its abundant growth factors and nutrients. A comparative clinical study conducted by Goyal *et al.* indicated that the utilization of PRP as a biomaterial for guided tissue regeneration in the treatment of apicomarginal defects resulted in significant clinical improvements compared to collagen scaffolds [223]. Furthermore, contrasting to other alternative substrates and scaffolds, PRP can act as serum to provide fundamental nutrients to support cell activities [224]. Therefore, many studies have employed RPR to provide a suitable cell microenvironment to reduce cell apoptosis and accelerate cell growth and thus boost uterine regeneration. In fact, PRP cocultured with human MenSCs confirmed that PRP enabled to promote cell survival rate and inhibit cell apoptosis [106]. It may be attributed to the ability of PRP to stimulate the Akt and IL-6/STAT3 pathways, leading to an increased proportion of cells in the S phase and consequently promoting MenSCs proliferation. These findings highlight the potential therapeutic application of activated PRP for uterine regeneration.

On the other hand, abundant growth factors in PRP can functionalize as a beneficial stimulus to regulate cell activities. Many studies have indicated that PRP can release growth factors to modulate cell fate through paracrine signaling. TGF, for example, is one important growth factor in PRP. The release of TGF-β1 from PRP resulted in enhanced cell proliferation and mitochondrial activity in periodontal ligament cells and osteoblasts, along with an upregulation of alkaline phosphatase activity [225]. Moreover, TGF-β1 could build a suitable regenerative microenvironment to reduce cell apoptosis and mitigate inflammation and immune response *in vivo*. IGF is a vital growth factor that participates in the uterine repair process. The proliferative and differentiating effects of IGF on uterine stem cells have been well-documented. Moreover, VEGF and FGF in PRP are another two crucial growth factors for angiogenesis and re-epithelization. The formation of blood vessels can assist the delivery of nutrients and oxygen, thereby providing a superior microenvironment for cell growth and tissue remodeling. VEGF and FGF are also essential for the proliferation of vascular endothelial cells and smooth muscle cells and thus can promote epithelization and glands formation. In this context, the criticality of multiple growth factors released from PRP for uterine regeneration cannot be overstated. Zhang *et al.* employed PRP as a cell carrier to treat IUAs in a rat model [179]. As a result, endometrium proliferation, angiogenesis, and morphology recovery were improved, collagen fibers and inflammation were decreased after the implantation of PRP cocultured with MenSCs. The improved regeneration of uterine tissues was mainly due to the paracrine restorative effect exerted by PRP. The growth factors and cytokines released from PRP stimulated MenSCs and other cells in the uterus to secrete IGF-1, SDF-1α, and TSP-1 (thrombospondin-1), thereby restoring the structure and functions of the endometrium and uterus. Moreover, the clinical trials uncovered that the injection of autologous PRP had the excellent therapeutic effect for the treatment of patients with thin endometrium [226,227]. PRP infusion could significantly increase the endometrium thickness over 7 mm and at the same time promote clinical pregnancy rates.

### 4.3 Biomaterial and scaffold-based strategies

To date, cell-based therapy has been the primary therapeutic approach for uterine regeneration. However, biomaterial and scaffold-based strategies also exhibit significant potential in treating uterine tissue defects by providing structural and biochemical support for cell activities and serving as carriers for biomolecules. Biomaterials can be divided into synthetic and natural biomaterials. The common characteristics of synthetic and natural biomaterials employed for uterine regeneration are their biodegradability and high biocompatibility.

#### 4.3.1 Synthetic biomaterials

**Fig. 7:**
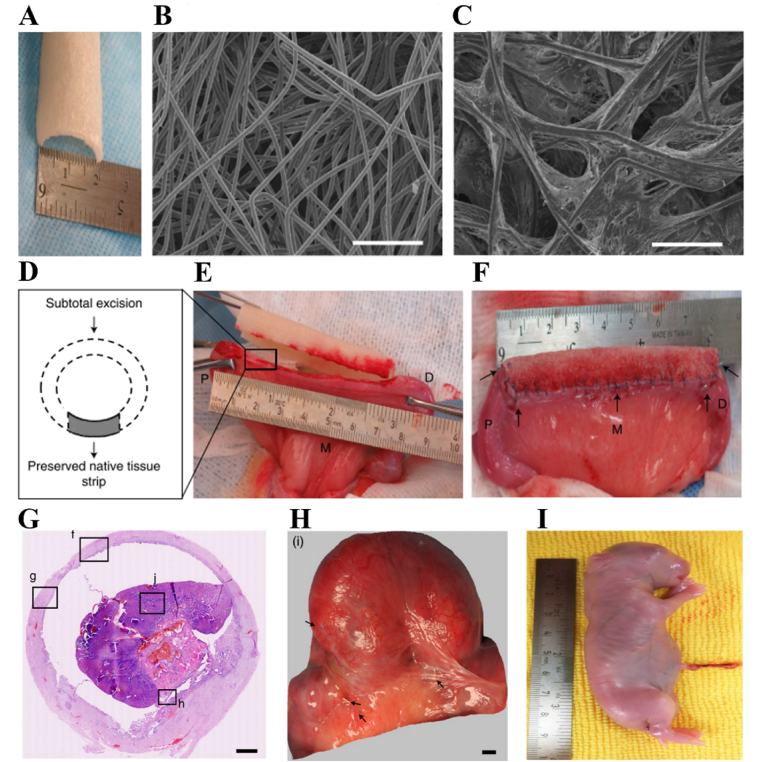
Biodegradable scaffold morphology and *in vivo* implantation. (A) Cross image of PLGA-coated PGA scaffolds. (B) SEM image of polymer scaffolds showing porous microstructure. (C) SEM image of cell-seeded scaffold *in vitro*. (D) Schematic illustration of subtotal uterine excision and scaffolds implantation process. (E) Surgical excision of one uterine horn and scaffold implantation. (F) The engrafted uterine horn. Black arrows represent tag sutures and titanium clips inserted at the anastomosis; P, proximal native tissue retained at the cervical end; M, mesometrium (mesentery to the uterus); D, distal native tissue retained at the tubal end. (G) H&E staining image showing cross-section of the pregnant bioengineered uterus. (H) The pregnancy of scaffold implantation group. (I) Gross appearance of a newborn from scaffolds implantation group. The scale bars in (B) and (C), 250 μm; (G), 2 mm; (H), 1 cm [241].

The mechanical properties and degradation rate of synthetic biomaterials can be readily manipulated by controlling the molecular weight and molecular structure [228]. Many synthetic biodegradable biomaterials, including polycaprolactone (PCL) [229,230], poly(glycolic acid) (PGA) [231,232], poly(lactic acid) (PLA) [233,234], PLGA [235,236], have been approved by FDA and massively used in tissue engineering and regenerative medicine. Therefore, synthetic biomaterials can play an essential role in uterine tissue engineering. PCL, a polyester approved by the FDA, is known for its thermal stability, biocompatibility, and low immunogenicity, making it easy to process and modify its surface. PCL is used in electrospinning to create nanofiber scaffolds that mimic the natural ECM for uterine tissue engineering. For example, melt electrospinning PCL meshes cultured with endometrium stem cells could be a potential therapy for vaginal wall repair [237]. Ultra-fine electrospun PCL fibers with slow degradation rate enhanced endometrium stem cells ingrowth. As a result, PCL composite constructs seeded with endometrium stem cells facilitated tissue integration and anti-inflammation response *in vivo*. PCL was also mixed with Pluronic F68 to prepare PCL/Pluronic F68 hybrid scaffolds incorporated mifepristone for the long-term treatment of chronic endometriosis [238]. Furthermore, electrospun PCL nanofibrous scaffolds conjugated with maltose could support uterine cell growth and would be a potential promise to treat uterine injuries and promote uterine regeneration [239]. However, PCL is hydrophobic, which can impede cell adhesion and limit its degradation rate. In addition to PCL, PGA was used to simulate three-dimensional architecture of endometrium tissue. PGA has good biocompatibility with reproductive tissues. Electrospun PGA scaffolds seeded with bovine endometrium epithelial cells and stromal cells were employed to bio-mimic 3D model of bovine endometrium, which might be beneficial for investigating endometrium pathophysiology in future studies [240]. Endometrium epithelial cells and stromal cells exhibited a hierarchical arrangement on PGA electrospun scaffolds and expressed positive vimentin and cytokeratin. However, PGA scaffolds have limitations, such as a high degradation rate and poor mechanical properties. PLGA, known for its biocompatibility and controllable biodegradability, is prominent in various tissue repair. But PLGA has limited mechanical properties. Therefore, PLGA can be combined with other materials to enhance its effectiveness. A recent study employed PLGA-coated PGA scaffolds seeded with autologous cells to build a tissue engineering uterus to restore uterine structure and functions in rabbits (Fig. 7) [241]. In contrast to previous studies predominantly employing a rat uterine defects model, the authors conducted their noteworthy investigations using rabbit uteri. The implantation of PLGA-coated PGA scaffolds seeded with autologous cells could finally reconstruct the damaged uteri and successfully restore fertility and give birth healthy offspring. Moreover, these synthetic polymers can be functionalized as micro/nanospheres to encapsulate/deliver biomolecules for improving uterine reconstruction. For example, 17β-estradiol can be encapsulated in PLGA microspheres for endometrium regeneration [167], and VEGF can be incorporated in PLA microparticles to promote angiogenesis and further facilitate uterine regeneration [242].

**Fig. 8:**
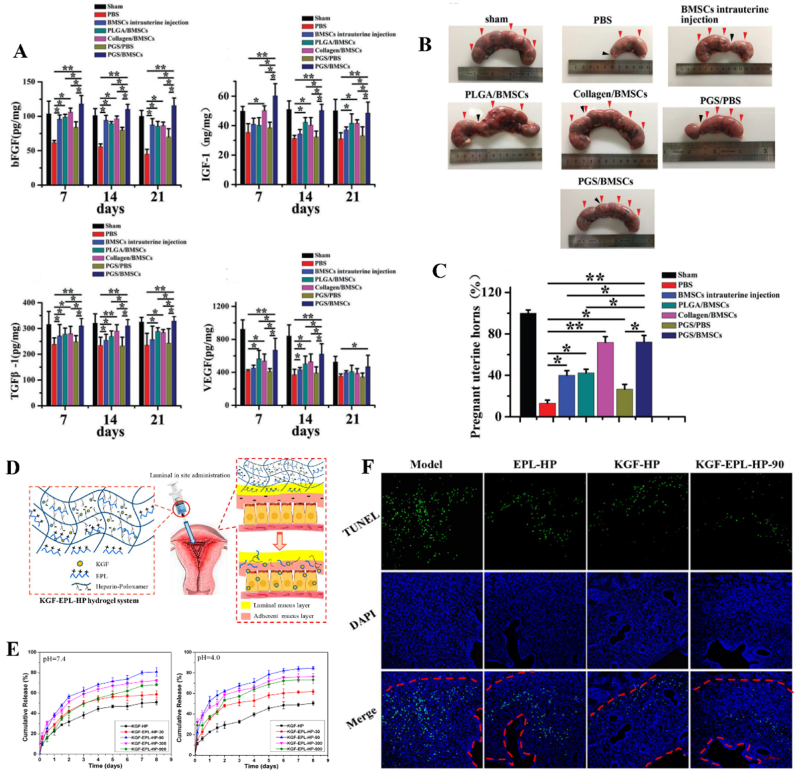
(A) The expression of bFGF, IGF-1, TGF-β1, and VEGF in injured uterus regions at different time points (7, 14, and 21 days) in shame, PBS, BMSCs intrauterine injection, PLGA/BMSCs, Collagen/BMSCs, PGS/PBS and PGS/BMSCs groups. (B) Representative images of embryo implantation for rats at 90 days post-operation in different groups. (C) Quantitative analysis of pregnancy rates in each group [103]. (D) Scheme of HP-EPL hydrogels loaded KGF for injured uterus healing. (E) *In vitro* sustained KGF release behavior from HP-EPL hydrogels with different EPL concentration in pH = 7.4 (PBS) and pH = 4.0 (acetic buffer) solution. (F) TUNEL assay kit analysis of cell apoptosis in injury endometrium at 3 days post-operation. Red line: the boundary of endometrium basal layer; blue: cell nuclei, DAPI; green: apoptosis cells [246]. (For interpretation of the references to colour in this figure legend, the reader is referred to the Web version of this article.)

In addition to commonly used synthetic polymers, novel polymer materials have been synthesized as promising alternatives for uterine regeneration. Elastic poly (glycerol sebacate) (PGS) scaffolds were employed as a novel construct for the delivery of BMSCs to mitigate IUAs. Xiao *et al.* synthesized and engineered PGS scaffolds via solvent casting and particles leaching [103]. The PGS scaffolds favored BMSCs attachment *in vitro* and prolonged the retention time of BMSCs after implanted in the damaged rat uterus. Furthermore, the comparative trial revealed that PGS scaffolds loaded with BMSCs exhibited significantly elevated levels of TGF-β1, bFGF, VEGF, and IGF in the injured endometrium compared to PLGA/BMSCs and collagen/BMSCs scaffolds, indicating enhanced morphological recovery and improved receptive fertility (Fig. 8A–C). Another original matrix material applied to prevent IUAs formation after endometrial injury is heparin-poloxamer (HP) hydrogels. The HP thermosensitive hydrogels, being biocompatible and biodegradable, have been widely employed as a drug delivery system for sustained drug release and prolonged therapeutic effects, thereby facilitating efficient tissue reconstruction [[243], [244], [245]]. HP hydrogels encapsulated 17β-estradiol not only addressed the limitations of E_2_, such as its poor solubility in aqueous solutions and low concentrations at damaged regions, but also enabled sustained release of estradiol at specific target sites. This approach enhanced the therapeutic effect by promoting endometrium regeneration through suppression of endoplasmic reticulum stress via activation of the PI3K/AKT and ERK1/2 signaling pathways [208]. Meanwhile, Zhang *et al.* also demonstrated the therapeutic potential of estradiol-HP hydrogels in preventing IUAs [166]. They indicated that the recovery of IUAs depended on the upregulation of kisspeptin via the activation of ERK1/2 and MAPKs p38 signaling pathways. Additionally, because of the dynamic environment in the uterus caused by rapid turnover of the endometrium mucus, which would lead to the poor retention and absorption of delivered drugs and agents, Xu *et al.* prepared HP-ε-polylysine (EPL) hydrogels incorporated keratinocyte growth factor (KGF) to overcome the drawbacks (Fig. 8D–F) [246]. HP-EPL hydrogels released KGF in a controlled manner. The proliferation of endometrium epithelial cells, glands as well as angiogenesis were significantly enhanced, and cell apoptosis was obviously inhibited with the treatment of HP-EPL hydrogels.

#### 4.3.2 Natural biomaterials

Compared to synthetic biomaterials, natural biomaterials usually exhibit excellent biocompatibility and biodegradation properties. Owing to the inherent advantages, the application of natural biomaterials for tissue engineering has gained tremendous interests [[247], [248], [249]]. Moreover, several natural biomaterials have been used for injured uterine healing. Table 3 concludes natural biomaterials mainly used for uterine regeneration. Currently, natural biomaterials are constructed to 3D scaffolds for seeding/loading stem cells and encapsulating biomolecules to promote cell proliferation, angiogenesis, epithelization and thus improve uterine regeneration.

**Table 3:** Natural biomaterials for uterine tissue engineering.

Biomaterial	Function	Ref
Collagen	Collagen scaffolds seeded with stem cells facilitated endometrium regeneration and restored fertility, Collagen binding with growth factors and cytokines with controlled delivery behavior regulated uterine regeneration, Collogen scaffolds loaded with bioactive glass promoted angiogenesis and repaired uterine injuries.	[101,109,172,173,[250], [251], [252], [253]]
Gelatin	GelMA hydrogels preformed as drug-loading vehicle and stem cells-loading platform to improve neovascularization and endometrium regeneration.	[69,[254], [255], [256]]
HA	HA preformed as biological barrier to separate uterine wall and thus prevented IUAs, HA served as therapeutic vehicle to load stem cells and biomolecules and induced macrophage immunomodulation, cell proliferation, and angiogenesis and thus improved uterine regeneration.	[64,[257], [258], [259], [260], [261], [262]]
Chitosan	Chitosan combined with IUDs for the prevention of IUAs, Chitosan-based scaffolds loaded SDF-1α for improving endometrium regeneration.	[163,263]

##### 4.3.2.1 Collagen

Collagen is mainly derived from the tendons, skin, cornea, and blood vessels of mammalian animals as well as marine organisms. Due to its crucial role as a structural component in the extracellular matrix (ECM), collagen has emerged as an exceptionally versatile biomaterial extensively employed in tissue engineering and regenerative medicine [250,264,265]. Collagen is such a hydrophilic, highly biocompatible, and biodegradable natural polymer with low immunity that the applications of collagen in wound repair and bone, cartilage, tendon, neuron regeneration have been widely accepted [[266], [267], [268], [269]]. Moreover, collagen has been prepared as fibers, membranes, hydrogels, and scaffolds ranging from one dimension to three dimension in tissue engineering because of its high flexibility. Compared to other natural polymers, collagen as a tissue framework exhibits excellent mechanical properties to support the 3D structure of cells, which is beneficial for cell growth and differentiation. For example, the collagen type I fibrils extracted from animal tendons possessed a strength of 120 MPa and an elastic modulus of 1.2 GPa due to its special triple helical structures [270]. Another attractive characteristic of collagen is that it can strongly bind to cell transmembrane receptors, such as integrins and discoidin domain receptors (DDR) via anchoring functions. Although the binding mechanism between collagen and cell transmembrane receptors is still not clear, the presence of anchoring functions is believed to facilitate cell attachment on collagen scaffolds and subsequently modulate cell activities to accelerate tissue reconstruction process. However, collagen has poor mechanical properties and undergoes fibroblast-mediated contraction following *in vivo* implantation, reducing its adhesion ability and absorbing quickly. Given its inherent advantages, collagen exhibits significant potential for regenerating uterine tissue [126,139,172,174,252,271].

**Fig. 9:**
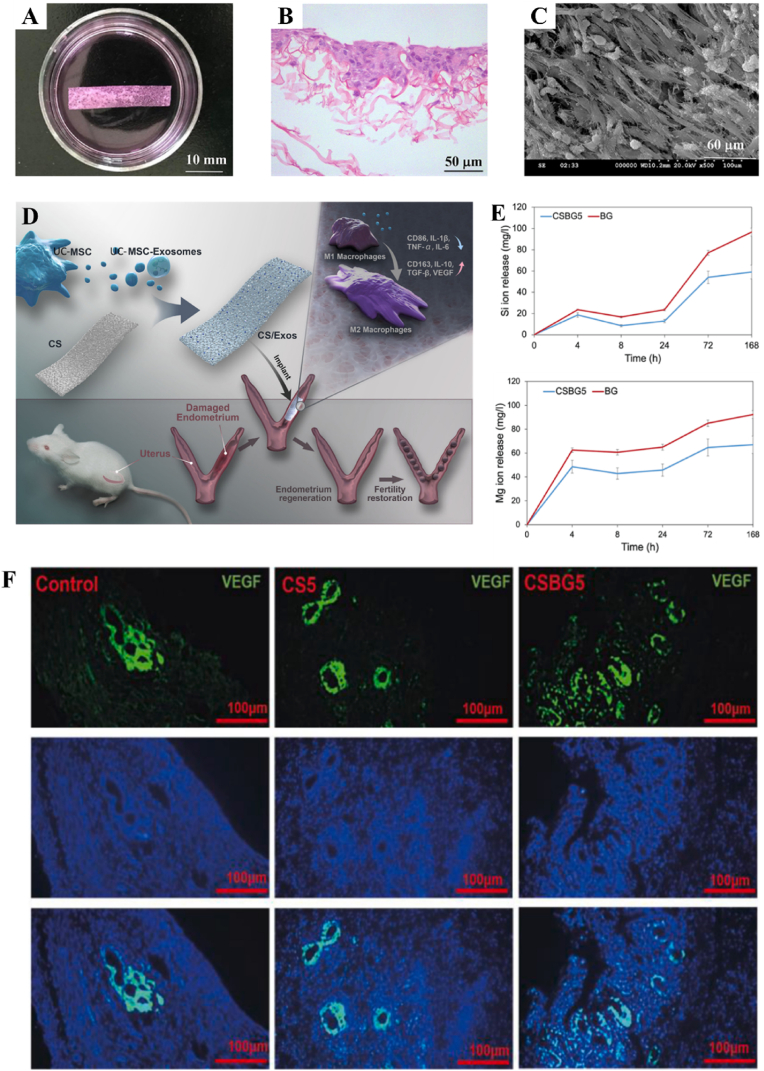
(A) Photo of a Type I collagen membrane loaded with human UC-MSCs. (B) H&E staining image of Type I collagen membrane loaded with human UC-MSCs. (C) SEM image showing human UC-MSCs on collagen membrane surface [109]. (D) Schematical illustration of collagen scaffolds laden with MSCs-derived exosomes for improving endometrium regeneration and restoring fertility via immunomodulation [277]. (E) Continuous silicate and magnesium ions released from collagen/borosilicate bioactive glass scaffolds. (F) The expression of VEGF after treating by collagen/borosilicate bioactive glass scaffolds [251].

There are two popular strategies applying collagen scaffolds for uterine regeneration. Combining collagen scaffolds with stem cell therapy is one potent approach. Collagen scaffolds cocultured with stem cells have been already demonstrated to be beneficial for regenerating and reconstructing various defective tissues and organs. For example, collagen hydrogels or scaffolds have been extensively investigated and utilized as carriers for osteoblasts or stem cells in bone tissue engineering for several decades [272]. While in the reproductive field, the application of collagen scaffolds loaded with stem cells for partial uterine regeneration or even the whole uterine reconstruction also gained increasing interests recently. The thought has been mainly carried out in a preclinical phase. MSCs, including BMSCs, UC-MSCs, and MenSCs are the major stem cells that emerge as promising cell types for uterine regeneration due to their multipotent differentiation, low immunomodulatory properties. Many studies have explored the possibilities of using collagen scaffolds loaded with these MSCs for uterine tissue engineering. Xin *et al.* reported that collagen type I membranes (0.5 % w/v) loaded with 50 μL human UC-MSCs (1 × 10^7^ cells/mL) could facilitate endometrium regeneration and restore fertility in a rate model [109]. Human UC-MSCs were cultured on collagen membrane to fabricate Collagen/UC-MSCs scaffolds for uterine tissue engineering (Fig. 9A–C). As a result, Collagen/UC-MSCs scaffolds improved human endometrium stromal cell proliferative rate and repress cell apoptosis *in vitro* via paracrine effect. The *in vivo* results suggested that Collagen/UC-MSCs scaffolds enabled to induce intrinsic endometrial cell proliferation and epithelium recovery and enhance the expression of ERα and PR, resulting in the improvement of embryo number and pregnancy rates. The efficacy of collagen scaffolds seeded UC-MSCs for the treatment of uterine defects was also investigated [273]. Moreover, there was a reported phase I clinical trial which used collagen scaffolds (4 cm × 6 cm) loaded with UC-MSCs (1 × 10^7^ cells) to treat patients with recurrent uterine adhesions [154]. A total of 26 patients suffering from recurrent IUAs were enrolled with a 30-month follow-up. Finally, 10 of them achieved pregnant, suggesting that collagen scaffolds seeded with UC-MSCs might be a safe and effective therapeutic method for treating IUAs. Another pilot study involving 18 infertile individuals with unresponsive thin endometrium showed that the endometrium thickness could be significantly improved after treated by collagen scaffolds (4 cm × 6 cm) seeded with UC-MSCs (2 × 10^7^ cells) [274]. 3 patients achieved pregnancy and 2 of them gave birth successfully. In addition to UC-MSCs, collagen scaffolds seeded with BMSCs were constructed for the functional regeneration of injured rate uterus. Collagen scaffolds with 3D architecture and porous structure built a superior microenvironment to support rat BMSCs attachment, proliferation, ingrowth, and differentiation. The collagen/BMSCs scaffolds expressed higher ratios of bFGF, IGF-1, TGFb1 and VEGF in wounded endometrium tissues, leading to increased proliferative ability to receive embryo implantation and development [139]. Besides, collagen scaffolds seeded with other stem cells, including human endometrium perivascular cells, bone marrow mononuclear cells, were employed to study the effect on repairing uterine injury in animal models, and their efficacy has been further demonstrated [101,126,275].

On the other hand, collagen scaffolds incorporating/binding biomolecules such as growth factors and cytokines are another attractive way for uterine regeneration. Pro-angiogenesis growth factors (e.g., FGF and VEGF) are primary choices for the endometrium or full-thickness uterine reconstruction due to their potent vascularization enhancement effect [276]. Li *et al.* demonstrated a potential strategy for regeneration of the uterine horn by employing collagen membranes (1.5 cm × 0.5 cm) loaded with human bFGF (8.4 μg) that specifically binds to collagen [173]. Collagen membranes loaded with bFGF by fusing a collagen-binding domain (CBD), TKKTLRT, to the N-terminal of bFGF via covalent reaction. Different from conventional drug loading methods, this binding strategy effectively restricted diffusion and prevented initial burst release following implantation, thereby extending the functionality of bFGF. The bFGF release rate primarily depended on the degradation of collagen scaffolds, which was good for newborn tissue ingrowth. The sustainable bFGF delivery system could facilitate uterine endometrium and muscle regeneration. Moreover, this strategy promoted the expression of vWF and enhanced vascularization and blood vessel density. Finally, the pregnancy rate (86.67 %) in collagen scaffolds loaded with collagen-binding bFGF was found to be comparable to that of normal uterus and significantly higher than the other groups. Inspired by the great success of collagen scaffolds loaded with collagen-binding FGF, a pilot study was conducted to investigate the impact of collagen-binding FGF on improving structural and functional reconstruction of scarred endometrium in 18 uterine infertile women [172]. Eventually, the results indicated the implantation of collagen scaffolds loaded with collagen-binding FGF (100 μg) led to significant improvements in menstrual blood volume, endometrial thickness, and scarred endometrial area among a majority of patients. VEGF is another commonly used pro-angiogenesis growth factor for repairing defective uterine tissues. Similarly, in terms of drug loading and controlled delivery, collagen-binding VEGF (5.56 μg) exhibited superiority over traditional physical diffusion methods [174]. The efficacy of collagen-binding VEGF was comparable to that of collagen-binding FGF. Except for growth factors, collagen scaffolds loaded with other cytokines such as Leukemia inhibitory factor (LIF) were also reported to promote the regeneration of rat uterine horns with full-thickness injury [177]. In addition to biomolecules, the utilization of collagen scaffolds loaded with 100 μL MSCs-derived exosomes (3 × 10^11^/mL) was employed for enhancing endometrium regeneration and restoring fertility [277]. As shown in Fig. 9D, the exosomes could facilitate CD163^+^ M2 macrophage polarization, reduce inflammation, and increase anti-inflammatory responses and thus promote uterine regeneration. Moreover, given the excellent angiogenic effect of bioactive glass, a recent study investigated the effect of collagen scaffolds encapsulated with borosilicate bioactive glass for the treatment of uterine injury [251]. Owing to the continuous release of magnesium and silicate ions, collagen scaffolds significantly improved angiogenesis and showed great potential for endometrium regeneration (Fig. 9E and F).

##### 4.3.2.2 Gelatin

Gelatin is a denatured protein derivative obtained by either acidic or alkaline hydrolysis of collagen. Gelatin is acknowledged as a generally-regarded-as-safe biomaterial by the FDA [278]. The clinical application of gelatin in tissue engineering and regenerative medicine, e.g., wound dressing [279,280], cartilage [281,282] and bone [283,284], neuron [285,286] regeneration, has been extensively investigated because of the unique advantages of gelatin: 1) gelatin is a denatured product that exhibits significantly reduced antigenicity and immunological reactivity compared to collagen; 2) there are quite a few arginine-glycine-aspartic (RGD) motifs in gelatin chains, which can be recognized by cell surface receptors to modulate cell adhesion, migration, proliferation, and differentiation [287,288]. It exhibits low toxicity, superior biodegradability, and biocompatibility, rendering it appropriate for diverse scaffolding methodologies. Gelatin preserves the biological activity of growth factors via affinity fixation and possesses adhesive characteristics that promote wound healing. Nevertheless, gelatin demonstrates instability at physiological temperatures and inadequate mechanical properties. These limitations can be mitigated through photo-crosslinking techniques to enhance the mechanical performance [289]. Therefore, gelatin is a potential promise for uterine regeneration [290].

**Fig. 10:**
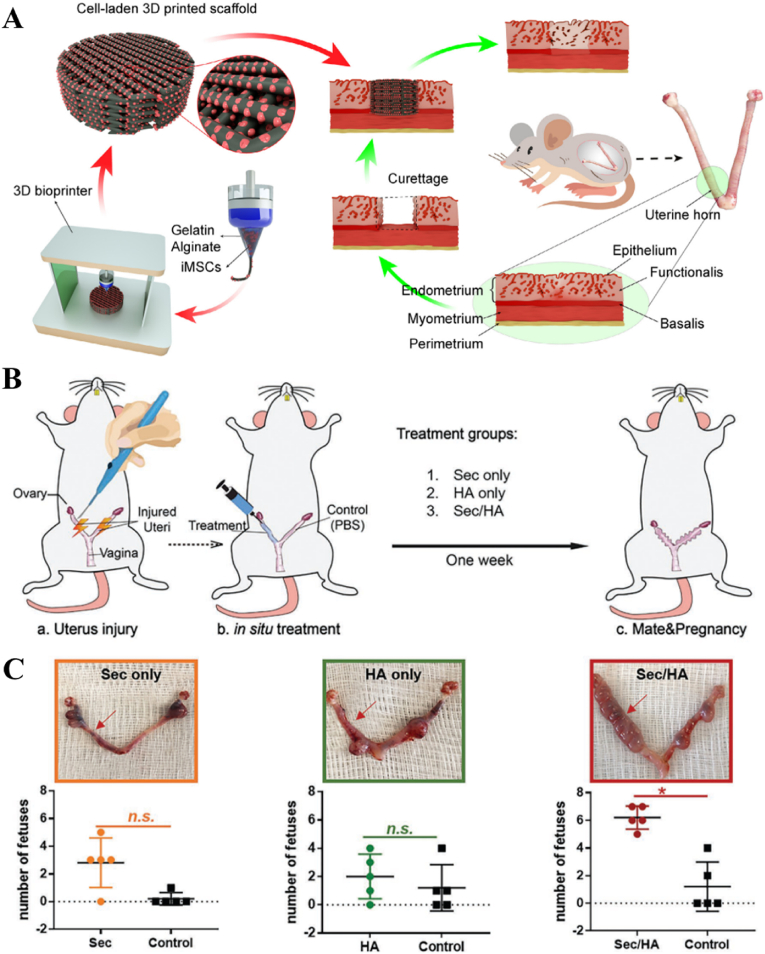
(A) Overall schematical illustration of cell-laden 3D printed gelatin hydrogel scaffolds for endometrium regeneration [256]. (B) Schematic illustration of animal study design. Both sides of the uterus are injured. One side gets treatment while left side gets PBS as control. And there are three treatment groups: MSC-Sec, crosslinked HA gel group; crosslinked HA group, and MSC-Sec group, n = 5. (C) Representative uterus images and quantitative data comparing the numbers of fetuses on both sides after treatment (red arrow indicates treated side). ∗ indicates P < 0.05 when compared to the other side [304]. (For interpretation of the references to colour in this figure legend, the reader is referred to the Web version of this article.)

A methacrylamide-functionalized gelatin (GelMA) hydrogel (5 wt% and 7 wt%) was prepared by Zambuto *et al.* to construct a tissue engineering platform for investigating endometrium angiogenesis and trophoblast invasion process in 3D microenvironment [291]. GelMA hydrogels could support the endometrium endothelial cell tube formation and endometrium stromal cell growth, indicating their potential as a tissue engineering model for the endometrium. In addition to biomimetic endometrium, GelMA could be employed as porous scaffolds to prevent severe IUAs. Cai *et al.* fabricated a new drug-loaded porous GelMA/Na-alginate (10 wt%/2 wt%) composite scaffold system prepared via microfluidic droplet template [255]. The GelMA composite scaffolds with a porous structure, resembling the shape of the uterine cavity, exhibited drug release capabilities that enhanced neovascularization and facilitated endometrial injury repair in a rat model. On the other hand, cell-laden gelatin and GelMA hydrogels have been widely used to accelerate soft and connective tissue remodeling [[292], [293], [294], [295]]. Gelatin and GelMA hydrogels co-cultured with various types of stem cells, such as BMSCs, MenSCs, UC-MSCs, etc., presented a promising approach for uterine tissue engineering. Unfortunately, there is a paucity of studies investigating the potential application of cell-laden gelatin and GelMA hydrogels in the reproductive field. So far, Ji *et al.* were the first to report on the pioneering use of cell-laden gelatin/alginate (10 wt%/1 wt%) hydrogels for uterine endometrium repair via 3D printing (Fig. 10A) [256]. In their study, 3D printed hydrogel scaffolds provided a suitable *in vitro* living environment for human induced pluripotent stem cell-derived mesenchymal stem cells (hiMSCs) (1 × 10^6^ cells/ml). The *in vivo* results implied the promotion of endometrium histomorphology recovery and regeneration of endometrium cells and epithelial cell. Moreover, the implantation of cell-laden hydrogel scaffolds significantly improved endometrium receptivity. Later, Chen *et al.* constructed a GelMA (10 wt%) and methacrylate sericin (SerMA) (5 wt%) hydrogel with human UC-MSCs (5 × 10^6^ cells/ml) encapsulation for repairing uterine injury [254]. As shown in Fig. 4C and D, UC-MSCs-laden hydrogels could increase endometrium thickness and promote endometrium regeneration.

##### 4.3.2.3 Hyaluronic acid

HA, also known as hyaluronan, is an anionic and non-sulfated glycosaminoglycan that exhibits widespread distribution in connective tissues of mammals. HA is an essential component of ECM and a multi-signaling molecule that can contact cell surface receptors such as CD44 to regulate cell behavior, including attachment, migration, proliferation, and differentiation [296]. It is a highly biocompatible and biodegradable nature derived polymer with a wide range of biological applications for wound healing, anti-inflammation, cancer prognosing, cartilage and neuron tissue engineering [[297], [298], [299], [300]]. However, HA has poor mechanical properties and low adhesion, and is difficult to remodel. In the uterus, HA serves as a modulatory cue to promote the trophoblast growth and invasion into the maternal endometrium via autocrine manner, thereby promoting successful maintenance of early human pregnancy [301]. Moreover, an *in vitro* study demonstrated that HA could effectively substitute albumin as the sole macromolecule in embryo cultures [302]. Consequently, HA exhibits great potential for uterine regeneration.

The functions of HA for the treatment of uterine defects can be also classified as two aspects. On one hand, HA functions as a biological barrier to separate uterine walls to prevent the formation of fibrosis, thereby suppressing IUAs. HA gel has been acknowledged as a biological intrauterine medical device because HA gel can not only provide important structural and mechanical support for the nearby cells but also perform biological functions to down-regulate inflammation response and promote angiogenesis [205]. Importantly, HA gel used as an intrauterine barrier has already been applied in clinical practice [54,73,75]. A series of clinical trials have demonstrated the safety and efficiency of HA gel for uterine regeneration. For example, Hooker *et al.* implemented a multi-center, prospective randomized controlled trial to examine the functions of auto-crosslinked HA gel on reducing the incidence of IUAs after dilatation and curettage (D&C) [77]. Although, the outcomes of 149 patients showed the down-regulation of the incidence and severity of IUAs as well as low mean adhesion scores, it was observed that HA gels did not significantly prevent the formation of adhesions. While another clinical study involving 48 patients indicated that the use of a new crosslinked HA gel was more likely to reduce the formation of adhesion in women undergoing curettage in the second trimester [258]. Furthermore, a comparative study has been conducted to further assess the safety and efficacy of this novel crosslinked HA gel in preventing IUAs [303]. The efficacy of HA gel was comparable to that of commercially available Lippes loop IUDs. Additionally, a recent meta-analysis of randomized clinical trials involving a total of 564 cases from 6 published studies assessed the clinical safety and efficacy of HA gels for the prevention of postoperative IUAs [257]. As a result, HA gels performed as a biological intrauterine barrier exhibited potential in preventing IUAs, particularly among individuals with lower adhesion scores and moderate severity. However, the use of HA gels as a standalone physical barrier proved insufficient in preventing severe IUAs. The application of HA gels alone for either partial or complete uterine regeneration remains unattainable.

On the other hand, an alternative approach involves utilizing HA hydrogels and scaffolds as therapeutic carriers for loading stem cells and bioactive molecules for uterine tissue engineering [260,261,304,305]. The natural half-life of HA gels when implanted *in vivo* is only 1–2 days due to the enzymatic environment in the mammalian uterus. Therefore, HA gel loaded with stem cells or growth factors cannot be directly implanted into the uterus if without crosslinking. Given the abundance of hydroxyl groups in HA, HA can be modified through esterification and methacrylate (MA) protonation to effectively decelerate the biodegradation rate of HA gels. For example, Liu *et al.* prepared crosslinkable HA (HAMA) gels [304]. HAMA gels loaded with mesenchymal stem cell-secretome (MSC-Sec) performed a sustained MSC-Sec release manner, thereby promoting the migration and growth of endometrial cells as well as tube formation of HUVECs. In addition, the sustained release of MSC-Sec from HAMA gels significantly enhanced endometrial functional restoration and improved pregnancy rates in rat models (Fig. 10B and C). Other alternative methods for gel preparation have been employed to facilitate the formation of HA gels. Fibrinogen/thrombin combined with HA hydrogels was fabricated to culture decidualized endometrial stromal cells for uterine damage repair [261]. The fibrin/HA hydrogels showed great biocompatibility *in vitro*. After implantation, fibrin/HA hydrogels loaded with EMSCs (1 × 10^7^ cells/ml) attenuated the area of fibrous tissues and increased the thickness of endometrium. The hydrogels facilitated the expression and secretion of biomolecules crucial for embryonic implantation, including Desmin (a muscle fiber protein), CD44, platelet endothelial cell adhesion molecule (PECAM), and IGF-1, resulting in an increased rate of embryo development.

##### 4.3.2.4 Other natural biomaterials

Although collagen, gelatin, and HA are widely used biomaterials for uterine regeneration, other natural biomaterials such as chitosan, silk, and cellulose have also emerged as innovative alternatives for the repair of injured uterus. Unfortunately, due to the relatively slow progress in uterine tissue engineering, there is a scarcity of annual research publications and data available. Consequently, only a limited number of publications have reported on the application of alternative biomaterials for uterine injury repair. Significant endeavors should be dedicated to the development of innovative and distinctive biomaterials for uterine regeneration in the future.

Chitosan is a natural polysaccharide which can be dissolved in an acidic solution and exhibits highly biocompatible and biodegradable and anti-bacterial properties [42,306,307]. Chitosan-heparin hydrogels have been reported to control the release of SDF-1α (2 μg) for preventing IUAs and facilitating endometrium regeneration [163]. The chitosan-based hydrogels with controlled and sustained drug delivery manner exhibited a remarkable ability to enhance endometrium thickness and increase the number of glands. Additionally, the hydrogels effectively reduced fibrosis area and most importantly promoted vascularization. Consequently, the chitosan hydrogels loaded with SDF-1α exhibited positive expression of CD 31 and CK 7, indicating enhanced regeneration of the injured endometrial epithelium and blood vessels. Moreover, chitosan nanoparticles loaded with antibiotics prepared via ionic gelation have been applied as transcervical drug vehicles to treat bacterial uterine infection [308]. The biocompatibility and water absorption properties of silk fibroin and cellulose, along with their ability to modulate cell behavior, make them well-suited for applications in tissue engineering and regenerative medicine [[309], [310], [311]]. Silk fibroin-cellulose membranes incorporated recombinant SDF-1α were constructed for the treatment of impaired uterus [164]. The damaged uterus resulted in improved endometrium regeneration and arteriogenesis formation, leading to enhanced pregnancy rates. Furthermore, oxidized cellulose was combined with HA to prevent adhesion reformation after adhesiolysis in rat models [312]. The results showed oxidized cellulose hydrogels were efficient for reducing adhesion formation *in vivo*.

#### 4.3.3 Decellularized tissues

Tissue-engineered decellularized ECM is an effective bio-scaffold for the repair and regeneration of defective tissue [[313], [314], [315]]. The safety and efficacy of decellularized tissues, i.e., dermis, blood vessel, heart valve, small intestine (SIS), amnion membrane, urinary bladder, from human and mammalian animals (mainly from cows and pigs) have been demonstrated in numerous clinical trials [[316], [317], [318], [319]]. Currently, there are several commercially available decellularized tissue products in the market, such as AlloDerm, CuffPatch™, Surgisis®, Oasis®, MatriStem, Pelvicol and Dura-Guard®. For example, a decellularized graft derived from the bovine SIS matrix has been successfully prepared for repairing critical-sized full-thickness skin defects in a small rodent model [320]. A prospective, randomized, double-blind, multi-center clinical trial involving a total of 196 patients has been conducted to evaluate the clinical efficacy of SIS for Lichtenstein hernioplasty [321]. Despite the widespread use of various decellularized ECMs in tissue regeneration, only a limited number of tissues or organs, such as the uterus and small intestinal submucosa, have been successfully decellularized and employed for fundamental research and preclinical studies pertaining to uterine regeneration.

**Fig. 11:**
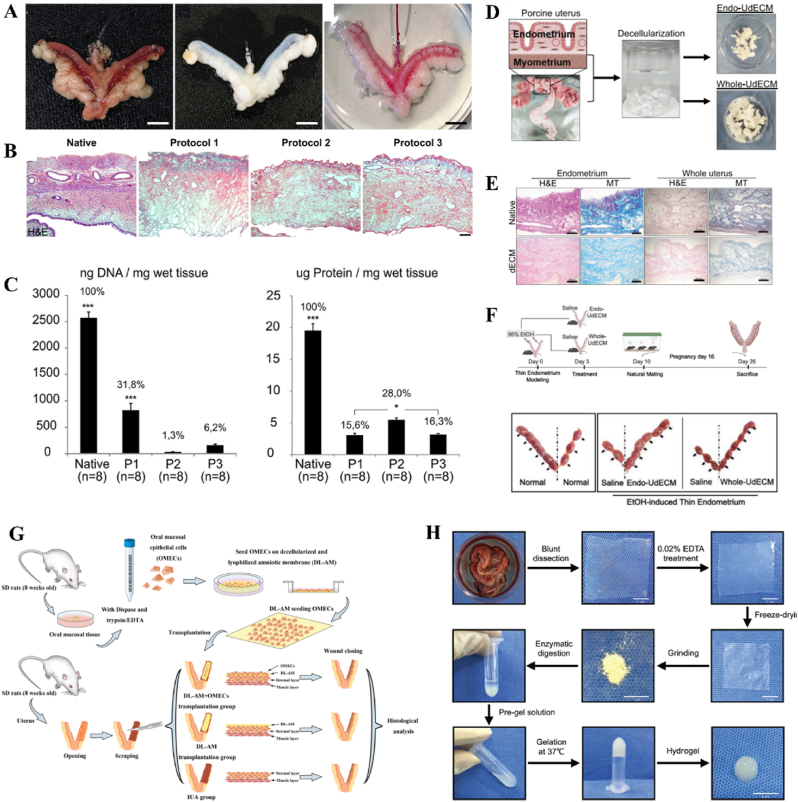
(A) The visual images of cadaveric native rat uterus, decellularized rat uterus, decellularized uterus by perfusion of a dye through the vascular system. scale bar; 1 cm. (B) H&E staining of native uterus, decellularization via perfusion with Triton X-100/DMSO/H_2_O (P1), decellularization via perfusion with sodium azide/dH_2_O (P2), and decellularization via perfusion with SDC/dH_2_O (P3). scale bar; 100 μm (C) DNA and protein quantification in native and decellularized uterus [324]. (D) Optical images of native and decellularized porcine endometrial and whole-uterine tissues. (E) H&E and Masson staining before and after decellularization process. scale bar; 500 μm. (F) Experimental illustration of fertility assessment and representative images of uteri with implantation sites in each group compared to normal mice. Black arrows indicate the implantation sites [328]. (G) The schematic illustration of decellularized amniotic membrane seeded with oral mucosal epithelial cells for injured endometrium regeneration [333]. (H) The process of preparing the injectable and thermo-sensitive hydrogels containing decellularized amniotic membrane [334].

The basic purpose of decellularization is to completely remove all cellular and nuclear contents from a tissue or organ while preserving the inherent ECM and vascular structure as well as biological functions via chemical and physical approaches [322]. The removal of cellular components and antigenicity from original tissues or organs is aimed at preventing disease transmission, reducing inflammation and immune response, as well as minimizing the risk of rejection following implantation into target sites. Meanwhile, the remaining decellularized ECM should maintain the native 3D architecture and microenvironment to provide mechanical support and biomolecular cues for modulating cell activities, including migration, attachment, proliferation, and differentiation. In this context, the optimal decellularization approach should effectively eliminate cellular materials while preserving the structural integrity of tissues and organs, as well as retaining essential biomolecular cues [323]. The selection of decellularization approaches can significantly affect the composition and structure of the obtained products, thereby influencing their biological functions post-implantation. Hellström and his co-workers compared three different decellularization protocols for rat uterine tissues: 1) perfusion with Triton X-100/dimethyl sulfoxide (DMSO)/H_2_O (P1); 2) perfusion with sodium azide/dH_2_O (P2); 3) perfusion with sodium deoxycholate (SDC)/dH_2_O (P3) [324]. As shown in Fig. 11A–C, although all three decellularization protocols effectively removed cellular content from uterine tissues, P2 and P3 demonstrated superior efficacy in DNA removal and preservation of ECM. Additionally, an alternative decellularized uterine tissue was constructed using three different perfusion protocols: 1) P1: perfusion with Triton X-100/DMSO/PBS; 2) P2: perfusion with Triton X-100/DMSO/dH_2_O; and 3) P3: perfusion with SDC/dH_2_O [325]. Notably, the pregnancy rates and fetal development observed in P1 and P2 outperformed those in P3, which contradicted the findings of Hellström's study.

Contrasting to natural and synthetic biomaterials, decellularized tissues and organs provide a specific niche for cell growth. The bioactive substances such as growth factors and glycosaminoglycans maintained after decellularization process enable to recruit surrounding cells to grow, proliferate, and differentiate and therefore can be beneficial for uterine regeneration after implantation. A whole rabbit uterus was decellularized to prepare tissue-specific ECM with a mild sodium dodecyl sulfate (SDS) perfusion [326]. The obtained decellularized ECM could be used to fabricate hydrogels to create a biomimetic environment for *in vitro* embryo development. Additionally, a decellularized scaffold derived from rat uterine tissues was surgically implanted into partially excised regions of the uterus to facilitate repair of defects in a rodent model [327]. Despite the slight aberration in the structure of decellularized uterine tissue scaffolds compared to that of a normal uterus, the results demonstrated their ability to support pregnancy in a manner comparable to an intact uterus. Yoshimasa *et al.* indicated that a decellularized uterine endometrium scaffold could induce epithelization and thus facilitate endometrium regeneration in rats [5]. Ahn *et al.* fabricated an injectable hydrogel containing porcine uterus-derived decellularized ECM for endometrium regeneration and fertility enhancement [328]. The decellularized ECM in their study exhibited excellent structural integrity and effectively preserved the majority of proteins and growth factors. As a result, the hydrogels created a conducive uterine environment for embryo development, leading to an increase in endometrium thickness and restoration of fertility upon injection (Fig. 11D–F). Although the decellularized uterine matrix exhibits potential in attracting and recruiting surrounding cells for uterine regeneration without prior seeding, researchers typically prefer to culture autologous or multipotent stem cells on a decellularized matrix to ultimately reconstruct patient-specific tissue for implantation. This process is known as recellularization. The utilization of recellularized uterine tissues following decellularization has emerged as a promising therapeutic strategy for tissue engineering uterus reconstruction, offering potential for uterine regeneration. The advantages for recellularization are that stem cells seeded on decellularized tissue scaffolds can provide new cell source and these cells enable to secrete beneficial growth factors and cytokines to stimulate injured tissues to grow and regenerate via paracrine effect. For example, Miyazaki *et al.* reseeded decellularized rat uterine matrix with adult and neonatal rat uterine cells and rat mesenchymal stem cells [329]. The recellularized uterine matrix reconstructed the structure and functions of injured uterine tissues and achieved comparable pregnancy rates to intact uterus.

In addition to decellularized uterus, decellularized tissues from other sources are also being engineered to regenerate defective uterine tissues and support pregnancy [330,331]. Although decellularized tissues from alternative sources may not serve as an optimal cellular niche for uterine-related cell activities, they do offer 3D structure and mechanical support for cell adhesion and preserve growth factors and glycosaminoglycans to modulate cell behavior. Most significantly, this approach effectively addresses the issue of limited availability of uterine sources by facilitating the utilization of easily accessible decellularized tissues such as SIS and amniotic membrane for the restoration of a defective uterus [332]. For example, Chen *et al.* conducted a study where they transplanted decellularized and lyophilized amniotic membranes seeded with oral mucosal epithelial cells into SD rats to facilitate the regeneration of injured endometrium (Fig. 11G) [333]. The bioengineered matrix performed great potential in preventing IUAs by improving uterine endometrium epithelium regeneration. Moreover, as shown in Fig. 11H, Li *et al.* constructed a thermo-sensitive hydrogel incorporating decellularized amniotic membrane, which effectively prevented endometrial fibrosis by the promotion of re-epithelialization of the damaged endometrium [334].

## 5 *In vivo* studies and clinical investigations

The damage or dysfunction of the uterus can potentially result in infertility, but it does not pose a threat to women's lives. Traditionally, surgeons prefer to perform uterus transplantation using donated healthy organs for patients who desire to conceive their own child. However, due to limited availability and potential immune rejection issues, most patients with AUFI are unable to receive this treatment. Fortunately, the emergence of tissue engineering strategies offers an alternative approach for restoring or regenerating the structure and functions of the uterus. Currently, some *in vivo* studies and clinical trials have demonstrated significant potential of cell-based, biomolecule-based, biomaterial and scaffold-based tissue engineering strategies in the reconstruction of uterine structure and functions. However, the progress in tissue engineering strategies for uterine reconstruction has been hindered by the relatively lower priority assigned to the human uterus compared to other vital organs such as bone, cartilage, skin, neurons, heart, kidney, liver, etc. Despite the limited available information on this topic thus far collected or reported upon, we have endeavored to provide a comprehensive summary of current *in vivo* studies and clinical applications pertaining to tissue engineering strategies for uterine regeneration.

**Fig. 12:**
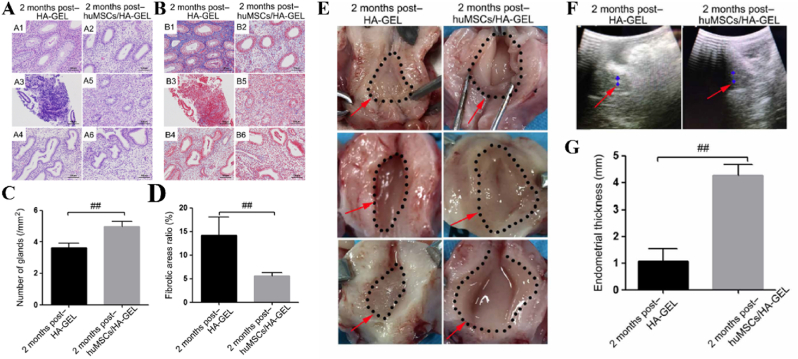
*In vivo* results in monkeys. (A) Endometrium H&E staining at 2 months after HA gels implantation and human UC-MSCs/HA gels implantation. (B) Masson staining of endometrium at 2 months after HA gels implantation and human UC-MSCs/HA gels implantation. (C) Number of glands per unit at 2 months after HA gels implantation and human UC-MSCs/HA gels implantation. (D) Fibrotic area ratio at 2 months after HA gels implantation and human UC-MSCs/HA gels implantation. (E) Representative images of uterine cavity at 2-month post-HA gels and post-human UC-MSCs/HA gels implantation. (F) Ultrasound detection of endometrium thickness in HA gels and UC-MSCs/HA gels group. (G) Endometrium thickness at 2 months after HA gels implantation and human UC-MSCs/HA gels implantation [305].

The application of tissue engineering strategies in uterine regeneration epitomizes the forefront of medical research. However, it is noteworthy that the current advancements are primarily limited to *in vivo* and preclinical investigations, predominantly employing murine or rat models as the preferred animal models for assessing the safety and efficacy of implanted stem cells, biomolecules, biomaterials, and scaffolds. To date, a variety of cells, biomolecules, and biomaterials (mentioned in Section 4) have shown great promise as efficient therapeutic agents capable of restoring and improving the structure and functions of the uterus within rat implantation models [[335], [336], [337], [338]]. However, the results obtained from murine or rat models, especially those related to efficacy, are often unreliable. Due to the robust regenerative capacity and limited endometrial thickness of these animals, positive outcomes may still be observed in cases where the extent of endometrial injury is insufficient, as they can automatically regenerate impaired sites. Therefore, it is imperative to use larger animal models such as rabbits, sheep, or even monkeys for further evaluation. Unfortunately, there have been limited cases where larger animal models were employed for safety and efficacy investigation. As aforementioned, a tissue-engineered uterus prepared by biodegradable PGA-PLGA hybrid polymers was successfully fabricated and subsequently seeded with autologous stem cells to restore and repair uterine structure and functions in rabbits [241]. Uterus transplantation is an acknowledged feasible treatment for patients with AUFI. In fact, compared with other parts in the whole uterus organ, endometrium matters a lot due to its dynamic regeneration capacity. Jones *et al.* tried to transplant endometrial tissues into rabbits with severe IUAs to restore fertility [339]. The improved pregnancy rate demonstrated the possibility of endometrium auto-transplantation for the treatment of IUAs in rabbits. Moreover, the significant progress of using BMSCs injection for prevention IUAs in rabbit models has been made [330,340]. Beyond murine and rabbit models, a recent study conducted an advanced investigation in rhesus monkeys [305]. Wang *et al.* used a complex of HA crosslinked gels and human UC-MSCs to partially repair IUAs caused by mechanical injury in monkeys. The effectiveness of HA gels as anti-adhesion barrier and the regenerative potential of human UC-MSCs on uterine tissues have been extensively demonstrated in numerous murine models and clinical applications [52,75,104]. However, in contrast to previous cases, this study represents the pioneering attempt to combine HA gels with human UC-MSCs for the treatment of severe IUAs in monkeys. As a result, the incorporation of human UC-MSCs into HA gels significantly increased glandular density and endometrial thickness while reducing fibrotic areas, thereby restoring both structural integrity and functional capacity within the uterine cavity (Fig. 12). In comparison to HA gels alone, the incorporation of human UC-MSCs on HA gels exhibited a remarkable improvement in endometrial reconstruction through paracrine effects. This was evidenced by the up-regulation of anti-inflammatory cytokines secretion alongside the down-regulation of pro-inflammatory cytokines.

Previous studies have extensively used small animal models, such as murine or rat models, to assess the safety and efficacy of uterine tissue engineering strategies. However, there is a paucity of clinical investigations pertaining to cell-based, biomolecule-based, biomaterial and scaffold-based tissue engineering for uterine regeneration. As shown in Table 4, it concludes the clinical trials of uterine tissue engineering strategies. Although some studies have been conducted to assess the feasibility of using cells, biomolecules, biomaterials and scaffolds for uterine regeneration in clinical practice, a comprehensive understanding regarding their safety and efficacy remains elusive. Cell-based therapy has exhibited significant safety and efficacy in treating uterine-related diseases based on murine and rat models [97,341,342]. However, when considering its clinical applications, there is a scarcity of resources available to substantiate its potential therapeutic efficacy on uterine regeneration. For example, although BMSCs, MenSCs, and UC-MSCs have been applied in some clinical trials for the regeneration of impaired endometrium and restoration of normal menstrual periods in women, the evaluation of their efficacy for uterine reconstruction remains challenging due to limited trial data. Additionally, the limited availability of preclinical studies renders other types of stem cells, such as ADSCs, currently unsuitable for clinical use. Hormone therapy, particularly for oral administration, is highly regarded in clinical treatment of biomolecules. Estrogen and progesterone enable to promote cell growth and formation of new blood vessels, but their low bioavailability and efficiency when administered orally remains a challenge. Encapsulation of hormones and other biomolecules in vehicles using tissue engineering strategies could potentially prolong therapeutic effects and improve uterine regeneration outcomes. However, the current advancements have only been observed in animal models. Further clinical trials are required to demonstrate the safety and efficacy of biomolecule-based therapy. On the other hand, the injection of PRP has been also clinically proved to be efficient for increasing endometrium thickness. PLA, PLGA, and PCL as desirable FDA approved biomaterials for tissue engineering have gained great clinical success in bone and cartilage tissue engineering, and natural biomaterials such as HA, collagen, chitosan, silk, cellulose, etc., are very popular in soft tissue regeneration [343,344]. However, the clinical utilization of biomaterial and scaffold-based strategies for uterine regeneration is in limitation. So far, only HA gels have been successfully used as physical barrier for antiadhesive purpose in clinical practice. The safety and efficacy of other biomaterials for uterine regeneration are rarely demonstrated in clinical trials.

**Table 4:** Clinical trials for uterine tissue regeneration.

Material and Cell	Year	Number of Participants	Clinical Outcome	Ref
Autologous bone marrow stem cells	2010	1	Endometrium thickness increased to 8 mm and *in vitro* fertilization and embryo transfer were done.	133
	2023	12	5 patients achieved pregnancy and 1 of them delivered a healthy baby.	345
CD133+BMSCs	2016	18	The average endometrium thickness increased to 6.7 mm, 3 patients became pregnant spontaneously, 7 pregnancies were obtained after 14 embryo transfers.	136
Collagen scaffolds + bone marrow mononuclear cells	2016	20	5 patients achieved successful pregnancies and live births.	101
MenSCs	2016	7	The endometrium thickness of 5 patients increased to 7 mm, 1 patient had spontaneous pregnancy and 2 patients successfully conceived after embryo transfer.	145
	2020	12	Endometrium thickness increased to 7.5 mm and the duration of menstruation increased to 5.3 days, 5 patients achieved pregnancy.	346
Endometrium stem cells	2014	6	Endometrium thickness increased to 5.48 mm, 5 patients resumed menstruation.	347
Collagen-binding bFGFs	2019	18	Endometrium thickness and blood vessel density were improved, 3 patients achieved pregnancy.	172
Collagen scaffolds + UC-MSCs	2018	26	10 patients became pregnant, and 8 of them delivered live babies.	154
	2021	16	The average endometrium thickness increased to 5.87 ± 0.77 mm, 3 patients got pregnant and 2 of them gave birth successfully.	274
PRP	2018	2	2 patients became pregnant.	348
	2018	20	12 patients achieved clinical pregnancy.	180
	2018	83	Endometrium thickness increased to 8.67 mm.	349
	2019	64	Endometrium thickness increased to 7.65 mm, 27.94 % patients achieved clinical pregnancy.	227
	2020	1	Vascularity and endometrium thickness were increased, The patient became pregnancy.	350
	2020	32	The endometrium thickness of 24 patients was 7 mm or thicker, 10 patients had clinical pregnancy.	226
	2021	30	No significant endometrium thickness increase and improved pregnancy rate were observed compared with standard treatment only.	351
	2021	54	27 patients achieved clinical pregnancy	352
HA	2013	107	Intrauterine balloon or intrauterine device was more effective than HA gel in the prevention of IUAs reformation.	353
	2015	90	HA gels could not prevent recurrence of IUAs and was not beneficial for pregnancy improvement.	354
	2017	152	HA gels could reduce the incidence and severity of IUAs but did not eliminate the process of adhesion formation completely.	77
	2018	564	HA gels could prevent IUAs, especially to those with moderate and low severity.	355
	2020	70	HA gels could significantly reduce the severity of IUAs.	356
	2020	89	HA gels had an advantage over an IUD in reducing IUA recurrence and decreasing adhesions.	357
	2021	272	HA gels might not improve IUA recurrence after hysteroscopic adhesiolysis.	67
	2022	47	The endometrium thickness, clinical pregnancy rate, and live birth rate was significantly improved.	68
	2022	200	Combination of HA gels and IUDs prevented the IUAs recurrence.	353

## 6 Challenges and outlook

Although the human uterus is not as indispensable for human survival as other vital organs such as the brain and heart, its damage or defects can significantly impair women's physical and mental well-being and prevent them from fulfilling their dream of parenthood. Therefore, uterine reconstruction should be given serious considerations. While uterus transplantation remains the most effective treatment for realizing patients' maternal aspirations, tissue engineering has emerged as a promising alternative therapeutic strategy for restoring, improving, and regenerating uterine structure and functions [358]. Increasing attention has been focused on uterine regeneration, particularly endometrial regeneration. Although significant progress has been made in reconstructing mammalian uteri using tissue engineering strategies to achieve endometrial regeneration and fertility restoration, there are still inevitable challenges that need to be addressed.

Table 5 summarizes the advantages and disadvantages of each strategy for endometrial or uterine regeneration. For example, stem cells derived from diverse sources have the ability to secrete ECM, cytokines, and growth factors via autocrine signaling, thereby attracting neighboring cells towards injured sites and accelerating endometrial or uterine reconstruction. However, the transplantation of these stem cells may result in low cell survival rates, thereby potentially hindering the regenerative process. In this context, 3D scaffolds made from either synthetic or natural biomaterials are used to incorporate stem cells to provide structural and mechanical support for cell activities while protecting cells from apoptosis. However, considering the remarkable elasticity of the uterus, capable of enduring multiple deformations during embryo development and delivery, previously fabricated scaffolds have failed to accurately simulate its highly stretchable properties. The modulation and promotion effect of biomolecules on uterine injury repair have been widely reported. Nevertheless, the acidic environment in the mammalian uterus restrains the use of a majority of biomolecules. Furthermore, it is imperative to develop controlled and sustainable in-situ drug delivery systems that effectively preserve the bioactivity of biomolecules. Sustainable in-situ release of biomolecule holds promise for mitigating burst release and achieving prolonged therapeutic effects compared to oral administration therapies.

**Table 5:** Advantages and disadvantages of different tissue engineering strategies.

Tissue Engineering Strategy	Advantage	Disadvantage
Cell-based	Providing cell sources to secrete ECMs, cytokines and growth factors and therefore attracting adjacent cells towards defective sites, Differentiating to specific cell lines to promote blood vessels formation and tissue regeneration, Modulating the host immune response.	Low cell survival rates and high cell usage amount *in vivo*, Possibility of ethical dilemmas and immune rejection.
Biomolecule-based	Recruiting and homing stem cells to defective sites, Regulating cell behavior, such as promoting cell proliferation and differentiation, reducing cell apoptosis and augmenting angiogenesis, Modulating inflammation responses.	Being easily inactive in acidic environment of the mammalian uterus, Unable to achieve sustainable and controlled in-situ release, Uncertainty regarding the dosage of drugs, such as orally administration of estrogen, Limited options.
Biomaterial and scaffold-based	Providing structural and mechanical support for cell activities, High flexibility in the selection of appropriate biomaterials (synthetic and natural) for regenerating defective tissues, Enabling to incorporate biomolecules for constructing sustainable release system.	Unmatched rate between biomaterials/scaffolds degradation and uterine tissues growth/ingrowth. Inability to simulate the highly elastic properties of uterine myometrium, Few choices for cell-laden 3D bioprinting.

As shown in Table 6, there exist five pivotal factors impeding the progress of uterine regeneration. Firstly, the current application of biomaterials, whether synthetic or natural, for endometrium or uterine regeneration is limited. Collagen and HA are the most commonly used biomaterials to repair endometrium defects and IUAs. Many *in vivo* studies have demonstrated that collagen and HA performed positive effect on uterine regeneration and the restoration of fertility. Moreover, several clinical trials revealed that the endometrium thickness was significantly increased in patients when treated by collagen and HA, leading to successful clinical pregnancies and the birth of healthy infants. However, besides collagen and HA, only a limited number of studies have investigated the potential applications of PLGA, PGA, chitosan, cellulose, etc., in the restoration and recovery of uterine structure and functions. Unfortunately, due to the limited extent of research conducted thus far, it remains challenging to validly assess the safety and efficacy of these biomaterials in uterine tissue engineering. Therefore, it is necessary to conduct more comprehensive animal studies as well as clinical trials in order to examine their potential in facilitating uterine regeneration in the future. On the other hand, numerous other biomaterials (e.g., polyurethane (PU), polyethylene glycol (PEG), alginate, carbon fibers, etc.) have been extensively investigated and applied in treating various elastic tissues such as muscle, blood vessel, gastrointestinal tract, but these excellent biomaterials have not been considered and used for uterine tissue engineering. For example, thermoplastic PU is an attractive biomaterial in tissue engineering for blood vessel, muscle, and nerve regeneration due to its excellent flexibility and high elasticity [359,360]. A recent study implied that PU elastomer scaffolds were significantly elastic and exhibited superior mechanical strength compared to native small diameter arteries. The *in vitro* results demonstrated the significant biocompatibility of the scaffolds. Furthermore, the *in vivo* implantation of PU scaffolds revealed visible neo-vessels, thereby providing further justifications for their potential application in blood vessel tissue engineering [361]. In addition to blood vessel regeneration, PU elastomer should be a promising biomaterial for uterine tissue engineering due to its excellent biocompatibility and remarkable elastic properties. Typically, the elongation of PU elastomer could be up to 500 %, surpassing that of native uterine tissue. Other new and innovative biomaterials should be further developed and made for uterine regeneration.

**Table 6:** Limitations and perspectives in uterine regeneration.

Limitation	Perspective
Limited biomaterials (e.g., collagen, gelatin, HA, PRP, PLGA, PGS, etc.) have been applied for endometrium or uterine regeneration.	Commonly used natural or synthetic biomaterials such as cellulose, chitosan, PU, PEG, etc., should be further explored for the repair of uterine defects.
Reconstruction of the full-thickness uterine (endometrium + myometrium) has been largely overlooked despite extensive investigations into endometrial regeneration.	More focus should be put on the full-thickness uterine reconstruction instead of endometrium regeneration. myometrium also matters a lot.
The endometrium thickness of murine and rat is very thin (much less than human beings), which could cause false positive results due to the insufficient mechanical injury and their robust regeneration ability.	Larger animal models (e.g., sheep, cattle, and monkey) with comparable endometrium thickness to humans should be performed to explore the safety and efficacy of tissue engineering strategies before clinical trials.
The application of tissue engineering strategies for uterine regeneration mainly stays in preclinical studies.	Preclinical studies should be comprehensive and clinical trials should be emphasized.
Most scaffolds have been fabricated via conventional scaffold fabrication methods.	3D bioprinting and 4D bioprinting are the promising approaches for the fabrication of scaffolds that integrate cells, biomolecules, and biomaterials to facilitate uterine regeneration.

Secondly, the current studies predominantly focus on constructing IUAs models to investigate and explore the efficacy of cell-based, biomolecule-based, biomaterial and scaffold-based strategies for regenerating endometrium layers. It is evident that the endometrium plays a crucial role in the human uterus and is closely associated with the release of sexual hormones during menstrual cycles and embryo development [362]. Endometrium usually possesses dynamic morphology and thickness. The severe damage to the endometrium layer caused by mechanical injury may cause infertility. However, the myometrium, consisting of smooth muscle cells and blood vessels, plays a vital role in supporting the uterine structure, facilitating oxygen and nutrient transportation, and accommodating significant deformations during embryo development. Moreover, it is important to acknowledge that gynecological diseases encompass not only endometrial injuries but also pose significant concerns regarding myometrium damage. For example, uterine leiomyoma is a benign smooth muscle tumor in the uterus that can result in pregnancy complications. Therefore, partial endometrial reconstruction is insufficient to address uterine-related diseases. In this context, researchers and surgeons should give priority to the full-thickness and whole uterine regeneration, including endometrium layer and smooth muscle layer reconstruction. While some studies have tried to repair the full-thickness uterine injury [126,174], the results were unsatisfactory due to the hierarchical and complex structures of the entire uterus involving different types of cells. More emphasis should be placed on achieving complete uterine regeneration, rather than solely focusing on the regeneration of the endometrium. It would be a significant breakthrough if a full-thickness uterus injury could be well treated. The myometrium is elastic and capable of enduring significant deformation during embryonic development and parturition. Therefore, in order to achieve the whole uterine regeneration, biomaterials and scaffolds should possess not only biocompatibility and bioactivity but also a high degree of elasticity. However, the utilization of currently available biomaterials with high elasticity in uterine regeneration remains limited.

Thirdly, it is worth noting that researchers tend to favor murine and rat models when investigating and evaluating the safety and efficacy of tissue engineering strategies for uterine defects repair. However, given their remarkably thin endometrial thickness and robust regenerative capacity, inadequate mechanical injury inflicted by researchers could potentially yield false positive outcomes. Additionally, the thin endometrial thickness observed in murine or rat models does not align with the typical human endometrial thickness, which typically exceeds 7 mm. The disparity in endometrium thickness between humans and preclinical animal models may contribute to potential failures. For example, HA gels have consistently demonstrated efficacy in inhibiting collagen fiber reformation and preventing the recurrence of IUAs in murine and rat models. However, clinical trials involving patients have revealed unfavorable therapeutic effects of HA gels for treating IUAs, with some studies even reporting its failure in severe cases. This failure can be attributed to a mismatch in endometrium thickness between animal models and humans. Therefore, lager animal models such as sheep, cattle, and even monkeys with comparable endometrium thickness to humans should be employed to evaluate the safety and efficacy of tissue engineering strategies for uterine regeneration. However, there is a significant lack of documented cases employing large animal models for uterine regeneration, thus presenting a substantial gap prior to clinical implementation.

Furthermore, as discussed in Section 5, the focus of tissue engineering strategies for uterine regeneration remains primarily on preclinical studies. Limited research has been conducted on clinical trials to investigate the safety and efficacy of cell-based, biomolecule-based, biomaterial and scaffold-based tissue engineering strategies. Before being used in clinical trials, comprehensive preclinical studies should be conducted to justify the safety and efficacy of tissue engineering strategies for uterine regeneration. In this context, more *in vitro* and *in vivo* studies are necessary to evaluate their effectiveness. Various animal models including murine, rat, rabbit, sheep, and even monkey should be employed to examine the therapeutic effect of tissue engineering strategies on uterine regeneration. Only if preclinical studies comprehensively investigate the safety and efficacy of tissue engineering strategies for uterine reconstruction can their applications in clinical trials begin.

Acellular 3D printing and cell-laden 3D bioprinting have been increasingly applied in tissue engineering and regenerative medicine [[363], [364], [365], [366], [367], [368]]. The future prospects of tissue engineering lie in the utilization of 3D printing technologies, as they offer customized design, rapid processing, and cost-effectiveness. Despite the great potential exhibited by 3D printing technologies in various tissue repair and regeneration fields, there remains a paucity of research investigating the utilization of 3D printed scaffolds for endometrium or uterine regeneration [369,370]. This may be attributed to a lack of emphasis on this particular area. Currently, Li *et al.* initially engineered 3D printed gelatin/alginate scaffolds encapsulated with human induced pluripotent stem cell-derived mesenchymal stem cells (hiMSCs) for the rat uterine endometrium repair [256]. In this study, 3D printed hiMSCs loaded scaffolds promoted the recovery of endometrium morphology and enhanced the proliferation of endometrium cells and epithelial cells, finally resulting in the success of pregnancy. Later, more studies were conducted to use 3D printed scaffolds to repair endometrium or uterine defects [[371], [372], [373], [374]]. However, apart from these limited investigations, there is a paucity of research regarding the utilization of 3D printing for uterine regeneration. Therefore, further studies are imperative to explore the potential application of 3D printing in uterine regeneration. Additionally, considering the intricate structural complexity and anatomical morphology of the mammalian uterus, 4D printing, where time is the fourth dimension, holds great promise as a prospective avenue for future research due to that 4D printed objects can change their shape and properties over time in response to suitable external/internal stimuli (e.g., temperature, pH, humidity and light), compared with the static objects fabricated by 3D printing. Despite extensive exploration of 4D printing in various tissue engineering fields such as bone and blood vessel, its application in uterine regeneration remains further investigation [375,376]. 3D and 4D printing technologies provide the possibility in integrating cells, biomolecules, and biomaterials, thereby paving a promising avenue for advancing uterine regeneration.

## 7 Concluding remarks

Uterine dysfunction resulting in AUFI has emerged as an inevitable gynecological concern for women, particularly among young patients with a strong desire for fertility. Generally, organ transplantation is an effective option to treat uterine-related diseases and therefore restore fertility. However, due to limited sources and potential immune rejection issues, alternative choices should be considered to restore, maintain, or improve uterine structure and functions. Conventional treatments such as hysteroscopic adhesiolysis and IUDs have been characterized as safe and effective therapeutic approaches in preventing endometrium adhesion recurrence and restoring normal uterine morphology in clinic. However, these strategies aimed at preventing endometrium adhesion are insufficient in meeting the need for complete endometrium regeneration or the partial/whole uterus reconstruction. As a result, tissue engineering strategies involving cell-based, biomolecule-based, biomaterial and scaffold-based have been developed as novel therapeutic approaches for the treatment of uterine defects. Despite the limited number of reported clinical cases, numerous preclinical studies have consistently demonstrated the great potential of tissue engineering strategies in reconstructing uterine structure and functions for fertility restoration. Based on previous knowledge and current advancements, it is evident that tissue engineering strategies involving cells, biomolecules, and biomaterials can effectively facilitate uterine regeneration. In summary, tissue engineering strategies provide a new perspective for uterine regeneration.

## CRediT authorship contribution statement

**Shangsi Chen:** Writing – original draft, Conceptualization. **James J. Yoo:** Writing – review & editing. **Min Wang:** Writing – review & editing, Supervision, Funding acquisition.

## Declaration of competing interest

The authors declare that they have no known competing financial interests or personal relationships that could have appeared to influence the work reported in this paper.

## Data Availability

Data will be made available on request.
